# A Heterodox Approach for Designing Iron Photosensitizers: Pentacyanoferrate(II) Complexes with Monodentate Bipyridinium/Pyrazinium-Based Acceptor Ligands

**DOI:** 10.1021/acs.inorgchem.5c00412

**Published:** 2025-04-01

**Authors:** Heiner Schmidt, Ramadan C. Oglou, Hüseyin O. Tunçer, Turkan G. U. Ghobadi, Şafak Tekir, Kubra N. O. Sertcelik, Abdelrahman Ibrahim, Lotta Döhler, Salih Özçubukçu, Stephan Kupfer, Benjamin Dietzek-Ivanšić, Ferdi Karadaş

**Affiliations:** † Department: Functional Interfaces, Leibniz Institute of Photonic Technologies, Albert-Einstein-Straße 9, 07745 Jena, Germany; ‡ Institute of Physical Chemistry, Friedrich Schiller University Jena, Helmholtzweg 4, 07743 Jena, Germany; § Interdisciplinary Nanoscience Center, Aarhus University, Gustav Wieds Vej 14, 8000 Aarhus C, Denmark; ∥ UNAM−National Nanotechnology Research Center, Bilkent University, 06800 Ankara, Türkiye; ⊥ Department of Chemistry, Main Campus, Bilkent University, 06800 Ankara, Türkiye; # NANOTAM−Nanotechnology Research Center, Bilkent University, 06800 Ankara, Türkiye; ∇ Department of Chemistry, Middle East Technical University, 06800 Ankara, Türkiye; ¶ Leibniz Institute of Surface Engineering, 04318 Leipzig, Germany

## Abstract

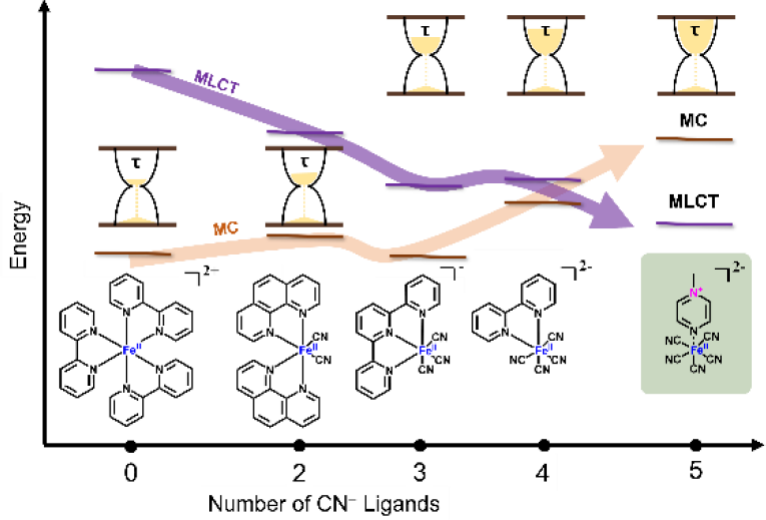

The main obstacle in replacing well-established precious ruthenium photosensitizers with earth-abundant iron analogs is the short excited state lifetimes of metal-to-ligand charge transfer (MLCT) states due to relatively weak octahedral field splitting and relaxation via metal-centered (MC) states. In this study, we address the issue of short lifetime by using pentacyanoferrate(II) complexes and combat facile photodissociation by utilizing positively charged pyrazinium or bipyridinium ligands. We utilize femtosecond transient absorption spectroscopy alongside quantum chemical calculations to probe the excited states of three 4,4′-bipyridinium- or pyrazinium-based pentacyanoferrate(II) complexes. The 4,4′-bipyridinium-based complexes exhibit ^3^MLCT lifetimes of about 20 ps, while the pyrazinium-based complex exhibits a lifetime of 61 ps in an aqueous solution, setting a benchmark for cyanoferrate complexes. These results mark the foundation for a new group of easy-to-prepare iron photosensitizers that can be used for harvesting visible light.

## Abstract 2

A high excited state lifetime of up to 61 ps in an aqueous medium can be achieved when pentacyanoferrate(II) group is connected to a pyridinium-based electron accepting group.

## Introduction

Earth-abundant photoactive complexes are critical for the advancement of light-driven molecular technologies, including dye-sensitized solar cells, artificial photosynthesis, and photocatalysis.^1−6^ One key focus is on Fe(II) complexes due to their isovalency with well-established precious d^6^ metal-based photosensitizers.^7−9^ The photoactivity of these complexes is governed by the competition between metal-to-ligand charge transfer (MLCT) and metal-centered (MC) states, the energetic position of which can be tuned by decorating the coordination sphere of the octahedral Fe(II) site with a combination of π-accepting and σ-donating ligands, including multidentate polypyridyl and N-heterocyclic carbene (NHC) ligands. This strategy has been successfully employed to develop iron PSs with excited state lifetimes in the order of hundreds of ps.^10−13^ Despite these exciting results, implementing an iron photosensitizer into a photocatalytic assembly remains challenging due to the synthetic difficulty in designing a multidentate ligand that can accommodate a catalytic unit. Furthermore, FeNHC complexes are sparsely soluble in aqueous media, limiting water splitting reactions to organic solvents.^14−21^ In this respect, an ideal ligand should not only increase the excited state lifetime of the iron PS but also coordinate efficiently to a catalyst for efficient charge separation.

**Figure 1:**
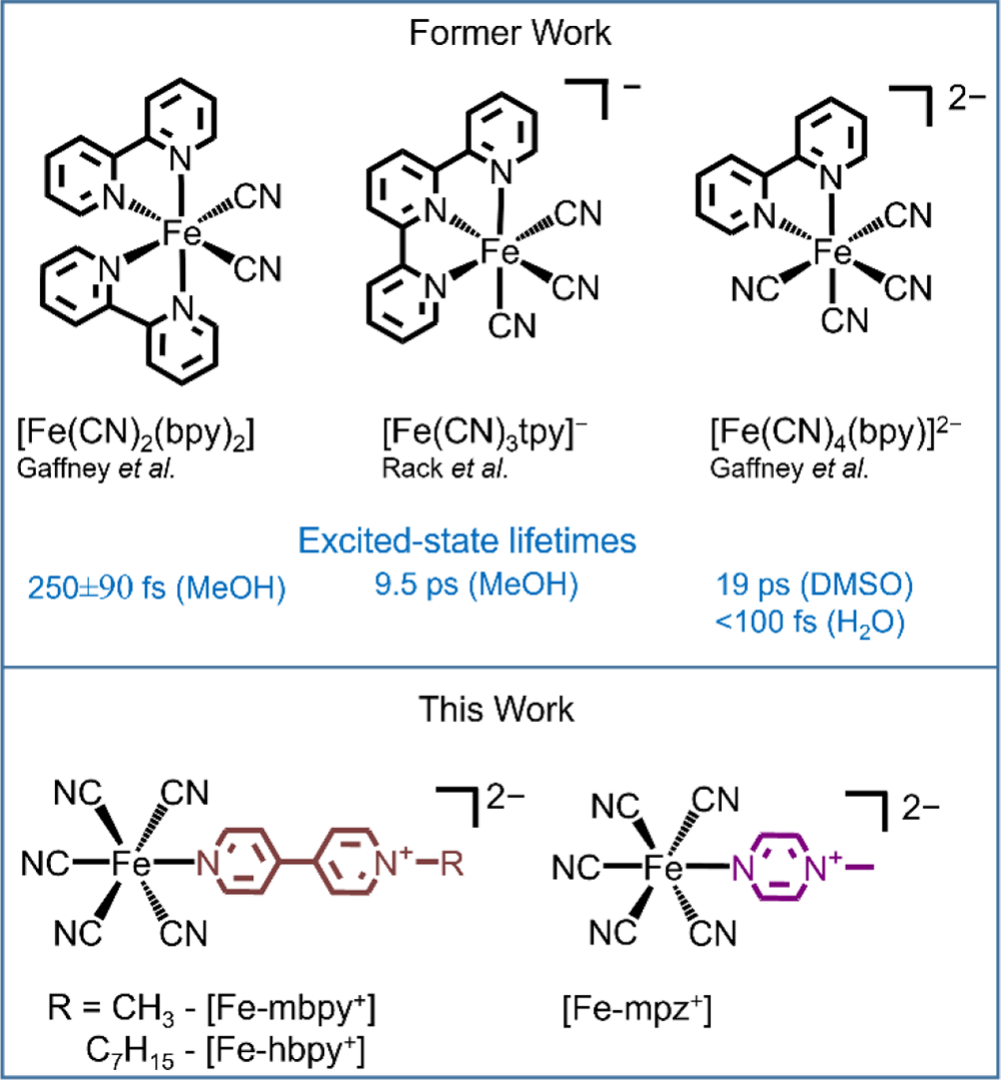
Chemical structures and excited-state lifetimes of previously studied Fe(II) cyano-polypyridyl complexes,^26,27,31^ alongside the complexes studied in this work.

The cyanide ligand offers attractive possibilities in this context, as it can serve as a short bridging ligand connecting metal ions by using both C and N atoms. Furthermore, it is a strong σ-donating ligand that can destabilize MC states, which hinders the rapid deactivation of the excited states in the corresponding complexes.^7,22^ Previous studies on polypyridyl-cyanoferrate complexes indicate that increasing the number of cyanide ligands around the Fe(II) site increases the MLCT lifetimes effectively by increasing the octahedral splitting, thus destabilizing MC states.^23,24^ The photophysics of dicyanoferrate complexes with two bidentate pyridyl ligands (Fe(L^L)_2_(CN)_2_),^25,26^ tricyanoferrate complexes with a tridentate ligand ([Fe(CN)_3_(L^L^L)]),^27^ and tetracyanoferrate complexes with a bidentate ligand ([Fe(CN)_4_(L^L)]^2–^)^28−31^ have been studied ((L^L) and (L^L^L) refer to bidentate and tridentate polypyridyl ligands, respectively). The photophysics of these complexes are mainly governed by competing deactivation pathways involving close-lying ^1,3^MLCT and ^3,5^MC states. The energy of the lowest-lying ^3,5^MC state rises significantly upon increasing the number of CN ligands: [Fe(bpy)_2_(CN)_2_] has an MLCT lifetime of 250 ± 90 fs (in MeOH)^28^ while it is 9.5 ps for [Fe(CN)_3_tpy]^−^ (in MeOH)^27^ and 19 ps for [Fe(bpy)(CN)_4_]^2–^ in aprotic solvents, with less than 100 fs in aqueous solutions (Figure 1).^32^ The lowest excited-state for the stated complexes is of ^3^MC or ^5^MC character. Furthermore, a recent computational study^27^ indicates that increasing the number of cyanide groups could be a feasible strategy to increase the energy of the lowest MC states in cyanoferrate complexes, which eventually leads to a switch of the MC and MLCT energy levels and in consequent to an elongation of the MLCT lifetimes. However, monodentate pyridyl-pentacyanoferrate (II) complexes have not been considered as potential Fe PSs due to their low photostabilities compared to bidentate and tridentate complexes.^33,34^

In this study, we report a strategy to overcome the low stability and to increase the lifetime of the MLCT by introducing cationic monodentate pyridinium ligands to afford water-soluble pyridinium-pentacyanoferrate(II) complexes and show that this design concept holds the potential to address all of the challenges mentioned above. To this aim, the involved photophysical properties are elucidated in-depth by a combination of steady-state and time-resolved spectroscopy and quantum chemical simulations.

## Experimental Section

Experimental details for synthesis and photophysics, alongside additional spectroscopic details, can be found in the Supporting Information.

(TD)DFT equilibrium structures, as well as high-resolution images, are freely available via Zenodo.^35^

## Results and Discussion

### Synthesis

Pyridinium pentacyanoferrate(II) complexes, *N*-methyl-[4,4′-bipyridin]-1-ium pentacyanoferrate(II) **[Fe-mbpy**^**+**^**]**, *N*-heptyl-[4,4′-bipyridin]-1-ium pentacyanoferrate(II) **[Fe-hbpy**^**+**^**]**, and *N*-methylpyrazinium pentacyanoferrate(II) **[Fe-mpz**^**+**^**]** were synthesized by reacting amminopentacyanoferrate(II) precursor **[Fe-NH**_**3**_**]** with the halide salts of respective ligands in aqueous solution. Further synthetic details alongside characterization via IR and NMR spectroscopy are summarized in Table 1 and in the Supporting Information (Figures S1–S7).

**Table 1:** Key Experimental Spectroscopic and Electrochemical Properties of the Aqueous Solutions **[Fe-NH_3_]**, **[Fe-mbpy^+^]**, **[Fe-hbpy^+^]**, and **[Fe-mpz^+^]**

					*E* vs Ag/AgCl, V^a^ (Δ*E*_P_, mV)^b^		
	ν_CN_	λ_MLCT,_ ε_MLCT_	λ_MLCT, TDDFT_	λ_π–π*_, ε_π–π*_	*E*_1/2_[Fe^II/III^] first oxidation (V)	*E*_1/2_[L^+^/L^0^] first reduction (V)	*E*_1/2_[L^0^/L^–1^] second reduction (V)
**[Fe-NH_3_]**	2032 cm^–1^, 2045 cm^–1^			225 nm (5.51 eV), 4000 M^–1^ cm^–1^	0.55 (37)		
**[Fe-mbpy^+^]**	2039 cm^–1^, 2051 cm^–1^	530 nm (2.34 eV), 4100 M^–1^ cm^–1^	523 nm (2.37 eV)	263 nm (4.72 eV), 20,700 M^–1^ cm^–1^	0.67 (67)	–0.62 (01)	–0.77 (10)
**[Fe-hbpy^+^]**	2039 cm^–1^, 2051 cm^–1^	530 nm (2.34 eV), 4300 M^–1^ cm^–1^		264 nm (4.70 eV), 19,000 M^–1^ cm^–1^	0.66 (73)	–0.55 (45)	–0.77 (88)
**[Fe-mpz^+^]**	2036 cm^–1^, 2051 cm^–1^	659 nm (1.88 eV), 11,900 M^–1^ cm^–1^	524 nm (2.36 eV)	277 nm (4.48 eV), 6400 M^–1^ cm^–1^	0.95 (15)	–0.44 (27)	

a 1 mM KCl solution with 0.1 mM analyte.

b Δ*E*_P_ = anodic and cathodic peak-to-peak difference

Upon coordination of the pyridinium ligands to the Fe(CN)_5_ fragment, symmetric stretching modes of CN shift from 2045 cm^–1^ for **[Fe-NH**_**3**_**]** to 2051 cm^–1^ for **[Fe-mbpy**^**+**^**]**, **[Fe-hbpy**^**+**^**]**, and **[Fe-mpz**^**+**^**]** due to the π-back-bonding from the Fe site to the ligand (Table 1, Figure S1). The effect of the electron-accepting ability of the pyridinium/pyrazinium ligands is also observed by the ^1^H NMR spectra, in which the peak at 8.93, 7.92, and 7.92 ppm for β-hydrogens (H-3&H-5) of mpz^+^, mbpy^+^, and hbpy^+^ ligands, respectively, shift to 8.18, 7.59, and 7.62 ppm once they are coordinated to the Fe(CN)_5_ core, indicating significant π-back-bonding (Figures S2–S7). These upfield shifts upon coordination of the iron complex are similar to those observed for metal-pyridyl complexes and the opposite of what is observed in protonated pyridine, where protonation causes a downfield shift compared to free pyridine.^36,37^

Cyclovoltammetry profiles (Figure S8) reveal a shift of the reversible Fe(II)/Fe(III) oxidation from 0.55 V (**[Fe-NH**_**3**_**]**) to 0.66 V for **[Fe-hbpy**^**+**^**]**, 0.67 V for **[Fe-mbpy**^**+**^**]** and 0.95 for **[Fe-mpz**^**+**^**]**. The first ligand reduction is obtained at a slightly lower potential for **[Fe-mpz**^**+**^**]** (−0.44 V) compared to **[Fe-hbpy**^**+**^**]** and **[Fe-mbpy**^**+**^**]** (−0.55 and – 0.62 V, respectively). The second ligand reduction for **[Fe-mbpy**^**+**^**]** and **[Fe-hbpy**^**+**^**]** occurs at −0.77 V.

### Photophysics

Steady-state absorption data for **[Fe-mbpy**^**+**^**]** and **[Fe-mpz**^**+**^**]** have been reported previously.^38−42^ Here, we report additional transient absorption data alongside quantum chemical simulations to elucidate the nature of the electronic transitions that govern the photophysical properties as well as the excited state relaxation pathways for **[Fe-mbpy**^**+**^**]**, **[Fe-hbpy**^**+**^**]**, and **[Fe-mpz**^**+**^**]**. As the spectra for **[Fe-hbpy**^**+**^**]** show strong similarities to **[Fe-mbpy**^**+**^**]**, we will focus the discussion on **[Fe-mbpy**^**+**^**]** and **[Fe-mpz**^**+**^**]**, while the data for **[Fe-hbpy**^**+**^**]** are compiled in the SI (Figure S9).

**Figure 2:**
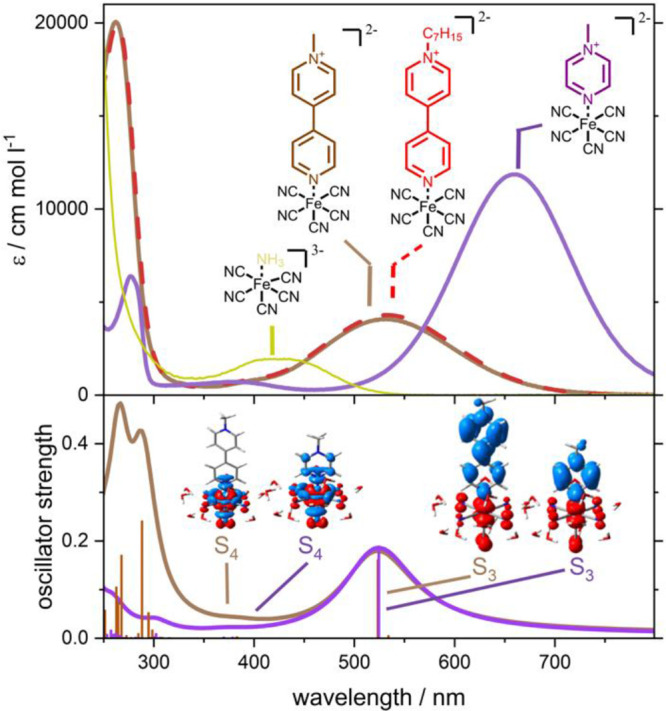
UV–vis spectra of **[Fe-NH**_**3**_]* (yellow), **[Fe-mbpy**^**+**^**]** (brown), **[Fe-hbpy**^**+**^**]** (red, dashed) and **[Fe-mpz**^**+**^**]** (violet). Subset shows the TDDFT calculated electronic absorption spectra of **[Fe-mbpy**^**+**^**]** (brown) and **[Fe-mpz**^**+**^**]** (violet). Strongly dipole-allowed transitions of **[Fe-mbpy**^**+**^**]** (brown) and **[Fe-mpz**^**+**^**]** (violet) into S_3_ states of ^1^MLCT character in the visible region are visualized by charge density difference plots; charge transfer takes place from red to blue. Weakly dipole-allowed ^1^MC transitions are predicted below 400 nm; for reference, a CDD plot for the transition into S_4_ (^1^MC) is shown. *The molar extinction coefficient for **[Fe-NH**_**3**_**]** is multiplied by 10 for visibility.

Before discussing the spectra of the pyridinium complexes, we first consider precursor **[Fe-NH**_**3**_**]** (Figure 2). **[Fe-NH**_**3**_**]** features a weak absorption at 425 nm with an extinction coefficient of just 193 M^–1^ cm^–1^, which can be attributed to weakly dipole-allowed MC transitions^42^ and more absorptions at below 225 nm with extinction coefficients of more than 4000 M^–1^ cm^–1^. **[Fe-mbpy**^**+**^**]** features absorption bands in the visible region, i.e., at 530 nm (extinction coefficient, ε = 4,300 M^–1^ cm^–1^) with a shoulder at 400 nm and an intense absorption in the UV region at 263 nm (ε = 20,700 M^–1^ cm^–1^). Similarly, **[Fe-mpz**^**+**^**]** shows a broad visible absorption centered at 659 nm (ε = 11,900 M^–1^ cm^–1^), as well as a weak absorption feature at 403 nm and an ultraviolet absorption at 277 nm (ε = 6400 M^–1^ cm^–1^). Closely related properties have been reported in the literature.^38^ According to density functional theory (DFT; B3LYP/def2-SVP) and time-dependent density functional theory (TDDFT) simulations, the underlying electronic excitations of the broad visible absorption bands stem from merely one singlet MLCT transition, i.e., from the molecular orbitals involved in the π-back-bonding between the Fe(II) center and the five cyanide ligands (π(*d*_*xz*_)) to the lowest energy π* orbitals of the mbpy^+^ and mpz^+^ ligands, respectively. The nature of the transitions is visualized by means of charge density difference (CDD) plots in Figure 2; further details can be found in Tables S1–S4. Notably, these quantum chemical simulations describe both explicit (e.g., hydrogen bonds) and implicit (e.g., polarization) solvent effects, i.e. typically known as microsolvation. To this aim, ten water molecules, which form the first coordination shell in the vicinity of the [Fe(CN)_5_]^3–^ moiety, and a polarizable continuum model have been combined (for more details concerning the computations, see the Supporting Information, all equilibrium structures as well as high resolution images are freely available via Zenodo).^35^

For **[Fe-mbpy**^**+**^**]**, a single dipole-allowed ^1^MLCT_mbpy_ excitation into S_3_ is predicted at 523 nm (2.37 eV), which is in excellent agreement with the experimental results of 530 nm. In the case of **[Fe-mpz**^**+**^**]**, a similar conclusion is drawn based on the quantum chemical simulations revealing a singly dipole-allowed transition in the visible spectral range, which is of ^1^MLCT_mpz_ character. However, this MLCT transition (into S_3_) is predicted with an almost identical excitation wavelength of 524 nm (2.36 eV) in contrast to the experimentally observed value of 659 nm (1.88 eV). Such a sizable overestimation of an MLCT excitation energy of 0.48 eV is rather surprising. We performed additional simulations using a *C*_S_ optimized structure of **[Fe-mpz**^**+**^**]** and merely an implicit solvent model to analyze the computational results further. In this case, the general picture remains the same: a single dipole-allowed transition associated with the same ^1^MLCT_mpz_ (into S_3_) is predicted at the TDDFT level of theory, while the excitation energy is 2.20 eV (564 nm) closer to the experimental. In addition, multiconfigurational simulations were performed using the restricted active space self-consistent field (RASSCF) approach. Unfortunately, a computationally affordable active space could not be constructed; see SI for details. Currently, we can only speculate that the deviation in excitation energy could be related to an insufficient description of (static) electron correlation as well as second sphere solvent effects or a combination of the aforementioned effects.

In general, we would expect a higher excitation energy for **[Fe-mpz**^**+**^**]** as it features a smaller conjugated π-system of six electrons in the pyrazinium ligand, while the π-system of **[Fe-mbpy**^**+**^**]** could potentially extend over 12 electrons in the bipyridinium moiety. Therefore, we conclude that the π-system of the two pyridine rings in an aqueous solution of **[Fe-mbpy**^**+**^**]** is partially disrupted, caused by a twist of both pyridine moieties with respect to each other, which hinders a fully constructive overlap of the respective π_mpz_/π_mpz_^*^ orbitals. This is in agreement with the crystal structure featuring a dihedral angle between the two aromatic rings of 21°^43^ as well as with the DFT-optimized structure of **[Fe-mbpy**^**+**^**]** in water (27°). In addition, the distance between the Fe core and the positively charged nitrogen atom is shorter in the case of **[Fe-mpz**^**+**^**]**, leading to a stronger electron-withdrawing effect and a lower MLCT energy for the mpz^+^-complex.

Further spectral signatures are present toward the UV region and stem from several weakly dipole-allowed ^1^MC transitions at 400 nm. These signatures appear as a shoulder at 400 nm for **[Fe-mbpy**^**+**^**]** and as weak absorption bands for **[Fe-NH**_**3**_**]** and for **[Fe-mpz**^**+**^**]** at 425 and 403 nm, respectively. In (pseudo)octahedral *d*^6^ complexes, typically, a set of six low-lying ^1^MC states (as well as six ^3^MC states) is observed. In the present Fe(II) complexes, the lowest ^1^MC states are associated with a reduced bond order between the iron and the aromatic coligand, which reflects the underlying electronic structure with an electron hole in a π(*d*) orbital and a singly populated σ_z2_^*^. Consequently, the σ bond along the *z*-axis with the aromatic ligand is most prone to (photo)dissociation.

At shorter wavelengths (below 300 nm) and in the case of **[Fe-mbpy**^**+**^**]** as well as of **[Fe-mpz**^**+**^**],** several strongly dipole-allowed intraligand (^1^IL) and ligand-to-ligand charge transfer (^1^LLCT) transitions are predicted at the TDDFT level of theory (see Tables S1–S4).

**Figure 3:**
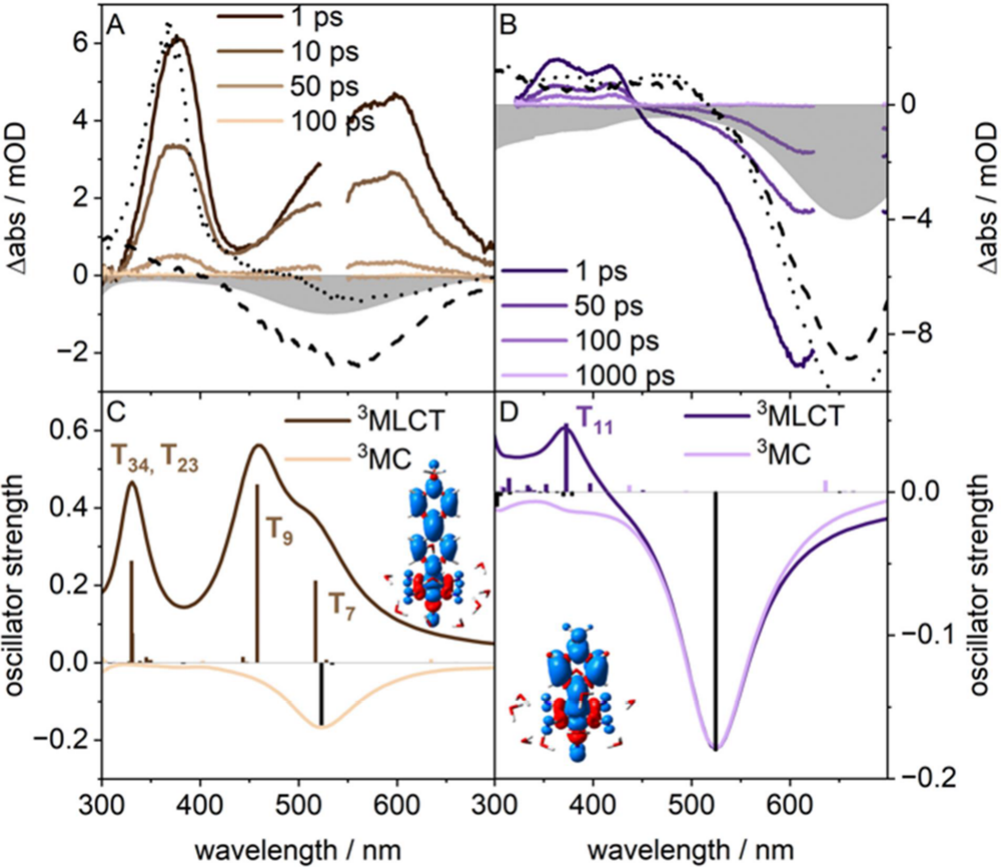
Experimental transient absorption spectra of aqueous solutions of **[Fe-mbpy**^**+**^**]** (A) and **[Fe-mpz**^**+**^**]** (B) excited at their respective ^1^MLCT (530 nm for **[Fe-mbpy**^**+**^**]**, 660 nm for **[Fe-mpz**^**+**^**]**. Simulated transient absorption spectra based on TDDFT calculations for the ^3^MLCT equilibrium (dark) and ^3^MC equilibrium (bright) are shown in panels C (**[Fe-mbpy**^**+**^**]**) and D (**[Fe-mpz**^**+**^**]**) alongside spin densities for the respective ^3^MLCT equilibria. Inverted steady state (gray area), reduced (dotted line), and oxidized (dashed line) are added as guides to the eye in panels A and B.

To understand the excited-state properties of the complexes, we employ femtosecond transient UV–vis absorption (TA) spectroscopy on **[Fe-mbpy**^**+**^**]**, **[Fe-hbpy**^**+**^**]**, and **[Fe-mpz**^**+**^**]** upon excitation of their respective MLCT transition at 530 nm (for **[Fe-mbpy**^**+**^**]** and **[Fe-hbpy**^**+**^**]**) and 656 nm (for **[Fe-mpz**^**+**^**]**) in combination with quantum chemical simulations (Figures 3 and S9–S13). UV–vis spectro-electrochemical (SEC) (Figure S14) measurements serve as a basis to simulate the absorption spectra of MLCT excited states.^44,45^

The transient absorption spectrum of **[Fe-mbpy**^**+**^**]** upon 530 nm excitation features an excited state absorption (ESA) at 380 nm and a broad asymmetric ESA band at 600 nm (Figure 3). The broad ESA at around 500 nm rises within the first 2 ps after excitation, and both ESA decay back to zero within 100 ps. The spectra resemble the reduced **[Fe-mbpy**^**0•**^**]** radical at low wavelengths as obtained from UV–vis SEC (see dotted line in Figure 3A), featuring absorption bands at 380 and 600 nm, and the structurally similar reduced methyl viologen at 390 and 600 nm.^46−48^

In order to rationalize the nature of the electronic transitions that contribute to the observed ESA and ground state bleach, we focused our computational analysis on two pathways associated with ^3^MLCT vs ^3^MC relaxation (Tables S5–S8). The relaxation from the ^1^MLCT to the ^3^MLCT state usually takes place within the first 30 fs after excitation, which is within the temporal resolution of our setup.^49−51^ To this aim, the lowest energy ^3^MLCT as well as the lowest energy ^3^MC states were preoptimized at the TDDFT level of theory and reoptimized using (unrestricted) DFT. Both DFT and TDDFT reveal consistent T_1_ character with comparable energies. Notably, TDDFT fails to provide meaningful ^3^MLCT energies in the absence of explicit solvent effects (see Figure S13 and Tables S9 and S10). Contributions of quintet states are not discussed in the following, as these are predicted by DFT to be fully dissociative and too high in energy. In the case of **[Fe-mbpy**^**+**^**]**, the simulated excited state absorption is modeled by the spin and dipole-allowed triplet–triplet transitions within the ^3^MLCT_mbpy_ (T_1_) state. This equilibrated ^3^MLCT state is predicted to exist at an energy of 1.34 eV. TDDFT reveals three transitions from this ^3^MLCT state within the spectral region of interest contributing to the ESA. The lowest two dipole-allowed transitions are obtained at 517 nm (2.40 eV) and 458 nm (2.71 eV) and are of ^3^IL (i.e., π*−π*; T_7_) and ^3^LMCT (T_9_) character according to TDDFT; see Figure 3C. Consequently, these two transitions form the characteristic broad ESA band observed experimentally as an asymmetric broad signal centered at 600 nm. Finally, the measured ESA signal at 375 nm can be related to higher-lying ^3^LMCT transitions (into T_23_) at 330 nm (3.75 eV). In addition, the spectral signature of the lowest energy ^3^MC state was evaluated computationally. To this aim, the lowest energy ^3^MC state was fully relaxed. Notably, this equilibrated ^3^MC state found at 1.00 eV is significantly lower in energy than the respective ^3^MLCT state. However, within the Franck–Condon point and the ^3^MLCT geometry, this ^3^MC state is localized at 2.5 and 2.7 eV. Thus, a considerable structural rearrangement reduces this state significantly in energy. In the present system, this is mainly associated with the partial cleavage of the Fe–N bond. Furthermore, the TA signal of the ^3^MC species was simulated (Figure 3C). Strikingly, TDDFT does not predict any dipole-allowed triplet–triplet excitations within the visible range. In fact, the lowest energy ESA signal is simulated at 283 nm (4.39 eV)–associated with a ^3^MLCT transition (into T_34_). Thus, the quantum chemical simulations clearly associate the ESA bands of **[Fe-mbpy**^**+**^**]**, with a ^3^MLCT state, as the second key species, which is of ^3^MC nature, does not show ESA within the spectral window of interest.

To understand the excited-state relaxation kinetics following excitation of the MLCT transitions in the visible spectral range, global analysis of the data is employed (see ESI Section 3.5 for more details). The fit reveals two processes for **[Fe-mbpy**^**+**^**]** (see Figure S10A), a fast process associated with a time constant of 0.9 ps (τ_1_) and a slower one with that of 18 ps (τ_2_). The former process is assigned to intra- and intermolecular vibrational relaxation based on lifetimes of similar [Fe(CN)_4_bpy]^2–^ complexes.^52^ Decay-associated spectra of the latter components resemble the reduced **[Fe-mbpy**^**0**^**]** at low wavelengths, as seen in the dotted line in Figure 3A- with both spectra showing a positive differential absorption at 375 nm. The ground state bleach (GSB) in the TA spectrum is underrepresented in the UV–vis SEC spectra. Therefore, and based on similar TDDFT calculated spectra showing positive features at 330 nm (^3^LMCT_mbpy_), at 458 nm (^3^LMCT_mbpy_), and at 517 nm (^3^IL), we assign the slower component to the decay of the ^3^MLCT with a lifetime of 18 ps.

The transient absorption spectra of **[Fe-mpz**^**+**^**]** feature two ESA bands at 360 and 400 nm and a broad GSB ranging from 420 to 700 nm. Similarly, as discussed for **[Fe-mbpy**^**+**^**]**, quantum chemical calculations were performed to address the energetics and spectral signatures of the lowest energy ^3^MLCT and ^3^MC states. The equilibrated ^3^MLCT state of **[Fe-mpz**^**+**^**]** is predicted at an energy of 1.05 eV. Thereby, the ESA bands between 360 and 400 nm can be assigned to several weakly spin- and dipole-allowed ^3^LMCT_mpz_ transitions, e.g. into T_11_ at 373 nm (3.33 eV; Figure 3D). As observed for **[Fe-mbpy**^**+**^**]**, no pronounced ESA within the equilibrated ^3^MC state (at 0.54 eV) is observed for **[Fe-mpz**^**+**^**]** based on the theoretical simulations (Figure 3D). Therefore, the measured TA signature reflects the electronic properties of the ^3^MLCT state. Similar to the case for **[Fe-mbpy**^**+**^**]**, global analysis for **[Fe-mpz**^**+**^**]** reveals a two-component decay. A faster component (τ_1_ = 13 ps) features weak ESA at 360 nm alongside two GSB signatures, one at 420 nm and a broad one from 440 until 700 nm, whereas the slower component (τ_2_ = 61 ps) features two ESA bands at 360 and 420 nm and a GSB centered at 600 nm. Differential UV–vis SEC spectra of **[Fe-mpz**^**+**^**]** feature similar spectral signatures, where both reduced and oxidized complex feature differential absorptions between 300 and 510 nm and bleach at 660 nm, corresponding to a loss of ground state MLCT. Therefore, and based on TDDFT calculations showing a blue positive feature at 373 nm alongside broad GSB in the red part of the spectrum, we assign the 61 ps component to the decay of the ^3^MLCT, which is substantially slower than for **[Fe-mbpy**^**+**^**]**. The observed difference in time constants for **[Fe-mbpy**^**+**^**]** and **[Fe-mpz**^**+**^**]** associated with the population of the relaxed ^3^MLCT states (τ_1_: 0.9 and 13 ps), as well as its radiationless decay back to the equilibrated singlet ground state (τ_1_: 18 and 61 ps), likely originate from a complex electronic situation with respect to the size of the ligand’s π-system and the charge localization of the positively charged nitrogen atom as well as the geometric changes between the relaxed ^3^MLCT and the Franck–Condon geometries. A root-mean-square analysis of the DFT-optimized structures (including the first explicit solvent shell) reveals a slightly larger rearrangement from the S_0_ to the ^3^MLCT geometry in the case of **[Fe-mpz**^**+**^**]** (0.0136 Å) in comparison to **[Fe-mbpy**^**+**^**]** (0.0102 Å). This more pronounced structural rearrangement in the case of **[Fe-mpz**^**+**^**]** is reflected by the longer τ_1_ value. Similarly, the radiationless decay of the thermally equilibrated ^3^MLCT back to the relaxed singlet ground state (τ_2_) is associated with the same geometry difference and the longer time component for **[Fe-mpz**^**+**^**]** in comparison to **[Fe-mbpy**^**+**^**]**. Notably, the vertical ^3^MLCT-S_0_ energy gap as obtained by (unrestricted and restricted) DFT within the ^3^MLCT structures is 0.86 and 0.77 eV for **[Fe-mbpy**^**+**^**]** and **[Fe-mpz**^**+**^**]**, respectively, rather similar and within the typical error range of DFT. Thus, these quantum chemical results suggest a comparable behavior of both compounds according to the energy gap law, while the larger structural rearrangement likely leads to the longer time components as observed for **[Fe-mpz**^**+**^**]**.

**Figure 4:**
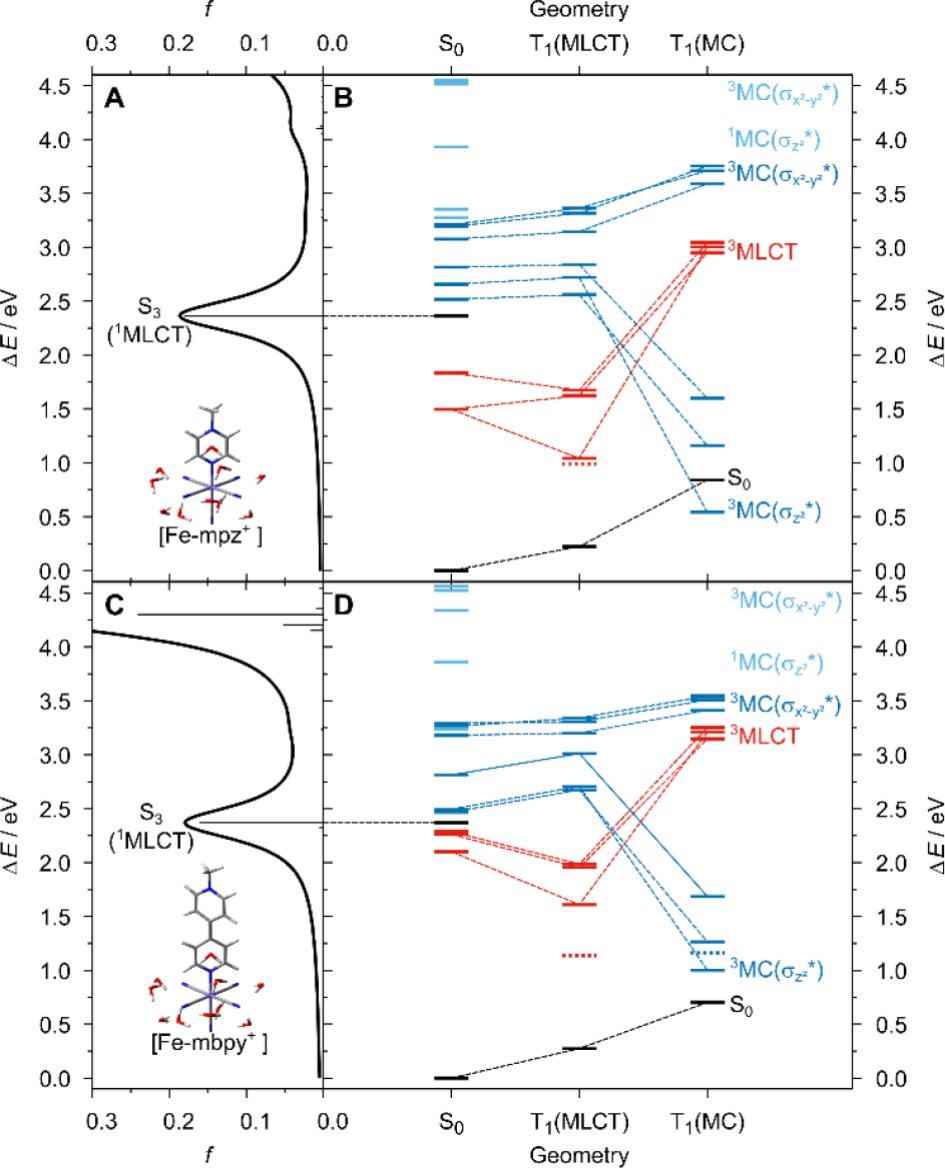
Simulated electronic absorption spectrum of **[Fe-mpz**^**+**^**]** (A) and **[Fe-mpz**^**+**^**]** (C) as obtained at the TDDFT level of theory (B3LYP/def2-SVP) in water (combined explicit-implicit water environment, i.e. microsolvation). B and D, excited state relaxation scheme involving the ground and excited state landscape within the fully relaxed S_0_, T_1_(^3^MLCT) and T_1_(^3^MC) geometries (from left to right). Singlet states are shown in black, ^3^MLCT states in red, and ^1^MC and ^3^MC states are visualized in light and dark blue, respectively. Unrestricted DFT energies of ^3^MLCT and ^3^MC states are displayed in dashed. All equilibrium structures as well as high resolution images are freely available via Zenodo.^35^

This unique sequence of electronic states has been investigated for **[Fe-mbpy**^**+**^**]** and **[Fe-mpz**^**+**^**]** within the three fully relaxed equilibrium structures of both Fe(II) complexes, namely, with the Franck–Condon geometry (S_0_) and within the ^3^MLCT and ^3^MC equilibria, see Figure 4.

Within the S_0_ geometry, the three ^3^MC states with the lowest energies are predicted to be well above the initially excited ^1^MLCT state and the three lowest ^3^MLCT states. Among the six ^3^MC states, the three ^3^MC states that are related to an excitation into the  orbital in an atomic orbital picture) are found significantly lower in energy than the ^3^MC states associated with a population of the  orbital (or ). This finding reflects–in complete agreement with the expectations–that the coordination bonds between the iron and aromatic ligands are comparably weak with respect to the Fe-CN bonds as the aromatic ligands are rather weak σ donors and π acceptors compared to cyanide ligands. In contrast, the five cyanide ligands with four of them within the *xy*-plane combine strong σ donor and π acceptor properties. Very similar results were obtained for the expected ^1^MC states, while these singlet states are observed at significantly higher energies, due to the sizable contribution of the exchange integral for spatially close electrons within the ^3^MC states. Upon ^3^MLCT equilibration, the three investigated ^3^MLCT states are stabilized by approximately 0.2 eV, while the ^1/3^MC states are destabilized. Only within the ^3^MC structure, the three ^3^MC_*z*^2^_ states are stabilized considerably, even to the level or slightly below the ^3^MLCT states within the ^3^MLCT structure. As discussed above, the large stabilization of the ^3^MC_*z*^2^_ states upon equilibration is associated with the weakening of the Fe–N bond in **[Fe-mpz**^**+**^**]** (S_0_: 2.003 Å, ^3^MC: 2.561 Å) and in **[Fe-mbpy**^**+**^**]** (S_0_: 2.054 Å, ^3^MC: 2.470 Å) and a partial dissociation of the aromatic ligand. Notably, unrestricted DFT (Figure 4B,C, dashed symbols) typically allows a more accurate description of the electronic structure in such stretched configurations in comparison to TDDFT. The population of the ^1/3^MC states can be clearly related to the photodegradation of the present Fe(II) complexes, while the main degradation channel proceeds with the loss of the respective aromatic ligand. Furthermore, the barrier along the ^3^MLCT-^3^MC pathway was estimated for **[Fe-mpz**^**+**^**]** and **[Fe-mbpy**^**+**^**]** at the unrestricted DFT level of theory. Unfortunately, the ten loosely bound water molecules hampered a full equilibration of the respective (triplet) transition states, see Supporting Information and Zenodo repository^35^ for details. However, approximate barriers of merely 0.20 and 0.22 eV were obtained between the fully relaxed ^3^MLCT and ^3^MC structures of **[Fe-mpz**^**+**^**]** and **[Fe-mbpy**^**+**^**]**, respectively; see Table S11. In consequence, these rather small barriers provide a fast deactivation pathway of the low-lying ^3^MLCT states via quasi-energetic ^3^MC states. Notably, this process is associated with a partial degradation within the coordination environment (i.e., Fe ··· N bond).

This behavior is verified in illumination experiments performed for **[Fe-NH**_**3**_**]**, **[Fe-mbpy**^**+**^**]**, and **[Fe-mpz**^**+**^**]** (see Figures S15–S17). Aqueous solutions were illuminated at the ^1^MC (405 nm for all complexes) and the ^1^MLCT transitions (590 nm for **[Fe-mbpy**^**+**^**]** and 660 nm for **[Fe-mpz**^**+**^**]**) with white light (5500 K LED). Photodissociation of monodentate ligands usually proceeds through the substitution of the monodentate ligand with solvent molecules.^48^ Photodissociation of **[Fe-NH**_**3**_**]** features multiple isosbestic points at 236, 255, and 378 nm, indicating the formation of one photoproduct. **[Fe-mbpy**^**+**^**]** and **[Fe-mpz**^**+**^**]**, however, feature drastic spectral changes after the first illumination cycles followed by continual changes (Figures S15B–G, S16, and S17). The illumination cascade for these molecules is likely more complex, where the initial photoproduct decays further. Quantum yields of photodissociation processes at the MC and MLCT excitations were calculated by using the real absorbed photon count based on the optical density at each time interval. Quantum yields for 405 nm excitation were determined as 32% for **[Fe-NH**_**3**_**]**, 0.04% for **[Fe-mbpy**^**+**^**]**, and 0.05% for **[Fe-mpz**^**+**^**]**. Photodecomposition after MLCT excitation proceeds quickly initially and slower later. Quantum yields in the first few time points were determined as 0.22% for **[Fe-mbpy**^**+**^**]**, excited at 590 nm and <0.1% for **[Fe-mpz**^**+**^**]**, illuminated with 660 nm and later as 0.08% for **[Fe-mbpy**^**+**^**]** and <0.01% for **[Fe-mpz**^**+**^**]**. Check Supporting Information for further information. Overall, photodissociation experiments reveal that **[Fe-NH**_**3**_**]** exhibits the highest photodissociation quantum yield of 32% at excitation at 405 nm due to the weak coordination of NH_3_ ligand to the Fe(II) site. **[Fe-mbpy**^**+**^**]** and **[Fe-mpz**^**+**^**]** exhibit far lower quantum yields of just 0.04 and 0.05%, respectively, at 405 nm due to the stronger coordination of cationic pyrdinium/pyrazinium ligands to the Fe(II) site. Excitation at the MLCT wavelength shows a quantum yield of 0.05 and 0.06% for **[Fe-mbpy**^**+**^**]** at 505 and 590 nm, respectively. **[Fe-mpz**^**+**^**]** exhibits a quantum yield of less than 0.1%, making the pyrazinium-based complex the most stable toward light excitation. The low photodissociation constants indicate that monodentate pyridinium-coordinated pentacyanoferrate(II) complexes hold the potential to be used in photocatalytic processes.

## Conclusions

We revisited the synthesis and characterization of three easy-to-synthesize Fe complexes bearing five cyanide groups and a monodentate pyridinium-based electron-accepting group. These complexes show excellent ^1^MLCT visible light absorption at 530 nm (**[Fe-mbpy**^**+**^**]**, **[Fe-hbpy**^**+**^**]**) and 660 nm (**[Fe-mpz**^**+**^**]**) and excited ^3^MLCT lifetimes of 18 ps (**[Fe-mbpy**^**+**^**]**), 21 ps (**[Fe-hbpy**^**+**^**]**) and 61 ps (**[Fe-mpz**^**+**^**]**), which is a record among cyanoferrate complexes. We find that the introduction of a cationic charge to the acceptor ligand and increasing the number of cyanide groups to five leads to an enhancement in the MLCT excited state lifetimes compared to Fe(II) cyano-polypyridyl complexes (of these complexes, [Fe(bpy)(CN)_4_]^2–^ exhibits the longest excited-state lifetime of 19 ps in aprotic solvents, which drops to less than 100 fs in an aqueous solution.^39^). TDDFT calculations show that the enhanced excited state lifetime originates from the lack of excited state relaxation through MC states since MC states lie energetically above MLCT states.

Our study not only highlights the feasibility of developing high-performance, earth-abundant iron photosensitizers but also provides a robust foundation for future research in solar energy conversion. Having water-soluble complexes at hand, water splitting can be performed directly in aqueous media, eliminating the need for organic solvents and allowing correlation between spectroscopic findings and catalytic activities. The integration of positively charged ligands represents a promising strategy for overcoming the inherent limitations of iron-based complexes, including the synthetic difficulty in connecting them to catalytic sites, which paves the way for the development of cost-effective and efficient solar energy technologies.

## Supporting Information Available

All calculated equilibrium structures and preoptimized transition state structures as well as high resolution images (charge density differences and spin densities) (PDF)
