# Supplementary material for: A Heterodox Approach for Designing Iron Photosensitizers: Pentacyanoferrate(II) Complexes with Monodentate Bipyridinium/Pyrazinium-Based Acceptor Ligands
Source: Inorg Chem. 2025 Apr 1;64(14):7079–87. doi: 10.1021/acs.inorgchem.5c00412 (PMC12001247; doi:10.1021/acs.inorgchem.5c00412)
Supplement: Supplementary file 1 — ic5c00412_si_001.pdf [file ic5c00412_si_001.pdf]

Supporting Information

A Heterodox Approach for Designing Iron Photosensitizers: Pentacyanoferrate(II) Complexes with Monodentate Bipyridinium/Pyrazinium-Based Acceptor Ligands

Heiner Schmidt,[a,b] Ramadan C. Oglou,[c,d] Hüseyin O. Tunçer,[e] Turkan G. U. Ghobadi,[f] Şafak Tekir,[g] Kubra N. O. Sertcelik,[e] Abdelrahman Ibrahim,[b] Lotta Döhler,[b] Salih Ozcubukcu,[g] Stephan Kupfer,*[b] Benjamin Dietzek-Ivanšić,*[a,b] Ferdi Karadaş*[a,b,d,e]

[a] Department: Functional Interfaces, Leibniz Institute of Photonic Technologies, Albert-Einstein-Straße 9, 07745 Jena, Germany

[b] Institute of Physical Chemistry, Friedrich Schiller University Jena, Helmholtzweg 4, 07743 Jena, Germany

[c] Interdisciplinary Nanoscience Center, Aarhus University, Gustav Wieds Vej 14, 8000 Aarhus C, Denmark

[d] UNAM – National Nanotechnology Research Center, Bilkent University, Ankara 06800, Türkiye

[e] Department of Chemistry, Main Campus, Bilkent University, 06800, Ankara, Türkiye

[f] NANOTAM – Nanotechnology Research Center, Bilkent University, 06800, Ankara, Türkiye.

[g] Department of Chemistry, Middle East Technical University, Ankara, 06800, Türkiye

Corresponding Author

karadas@fen.bilkent.edu.tr

benjamin.dietzek@leibniz-ipht.de

stephan.kupfer@uni-jena.de

1. Table of Contents

1. Table of Contents 2
2. Synthesis 3
2.1 Synthesis of *N*-methyl-[4,4'-bipyridin]-1-ium iodide, $mbpy^+I^-$ [1] 3
2.2 Synthesis of *N*-methylpyrazinium Iodide, mpz^+I^-[2] 3
2.3 Synthesis of $Na_2[Fe(CN)_5(C_{11}N_2H_{11})]\cdot xH_2O$ - [Fe-mbpy$^+$], $Na_2[Fe(CN)_5(C_{12}N_2H_{22})]\cdot xH_2O$ - [Fe-hbpy$^+$], and $Na_2[Fe(CN)_5(C_5N_2H_7)]\cdot xH_2O$ - [Fe-mpz$^+$] 3
3. Experimental Procedures 3
3.1 Chemicals 3
3.2 Material Characterization 3
3.3 Electrochemistry 4
3.4 UV-Vis SEC 4
3.5 fs-TA 4
3.6 Photodecomposition experiments 5
4. Computational Details 6
5. Results and Discussion 7
5.1 IR spectra 7
5.2 NMR 8
5.3 Electrochemistry 14
5.4 Transient absorption 15
5.5 Quantum chemical results 17
5.5 UV-Vis SEC 24
5.6 Photodecomposition 24
6. References 26
7. Author Contributions 26

2. Synthesis

2.1 Synthesis of *N*-methyl-[4,4'-bipyridin]-1-ium iodide, $mbpy^{+}I^{-}$ [1]

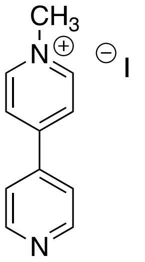

4,4'-Bipyridine (2.51 g, 16.0 mmol, 1 eq.) was dissolved in 50 mL acetone and allowed to be stirred until it was dissolved. Then, iodomethane (1.20 mL, 19.2 mmol, 1.2 eq.) was added dropwise and stirred at room temperature for 24 hours. After that, the precipitated product was filtered and washed several times with acetone and allowed to dry at room temperature. The product was obtained as a yellow solid (3.08 g, 10.4 mmol, 65% yield). ^{1}H NMR spectrum is consistent with the literature.[1]

2.2 Synthesis of *N*-methylpyrazinium Iodide, $mpz^{+}I^{-}$ [2]

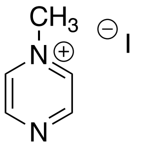

Pyrazine (2.00 g, 25.0 mmol) was dissolved in 10 mL iodomethane with a stirring bar at room temperature and allowed to rest without stirring while preventing light. 72 hours later, the crude product was obtained as a yellow solid. The reaction mixture was filtered, and further washing was done with the ethyl acetate (3x 30 mL). The product was obtained as a yellow solid. (4.46 g, 20.1 mmol, 80% yield). ^{1}H NMR spectrum is consistent with the literature.[2]

2.3 Synthesis of $Na_2[Fe(CN)_5(C_{10}N_2H_8CH_3)]\cdot xH_2O$ - [Fe-mbpy^{+}], $Na_2[Fe(CN)_5(C_{10}N_2H_8((CH)_2)_6CH_3)]\cdot xH_2O$ - [Fe-hbpy^{+}], and $Na_2[Fe(CN)_5(C_4N_2H_4CH_3)]\cdot xH_2O$ - [Fe-mpz^{+}]

Sodium amminopentacyanoferrate(II) has been prepared according to our previous method.[3] The synthesis of [Fe-L^{+}] has been performed according to previous literature[4]. An aqueous solution of 2.5 mL 1 mM pale-yellow colored sodium amminopentacyanoferrate(II) **[Fe-NH$_3$]** ($Na_3[Fe(CN)_5NH_3]\cdot 3H_2O$) was mixed with an aqueous solution of 2.5 mL 1 mM pale-yellowish colored L+ (mbpyI, hbpyBr, mpzI) ligand for 6 hours. Then 30 mL ethanol was added, and the solution was stored at 2 °C. To further purify the product, three more recrystallization steps were carried out with respective concentrations. The powder was dried at 60 °C to obtain pure [Fe-L^{+}] salts.

Elemental Analysis:

Calcd for **[Fe-mbpy^{+}]**, $Na_2FeC_{16}H_{11}N_7\cdot 6H_2O$: C, 37.59; N, 19.18; H, 4.53. Found: C, 37.79; N, 19.22; H, 4.32.
Calcd for **[Fe-hbpy^{+}]**, $Na_2FeC_{22}H_{23}N_7\cdot 4H_2O$: C, 47.24; N, 17.53; H, 5.59. Found: C, 47.82; N, 17.52; H, 5.44.
Calcd for **[Fe-mpz^{+}]**, $Na_2FeC_{10}N_7H_7\cdot 5H_2O$: C, 28.79; N, 23.51; H, 4.10. Found: C, 28.68; N, 23.83; H, 3.68.

^{1}H-NMR

[Fe-mbpy^{+}]; ^{1}H NMR (400 MHz, D_2O) δ 9.18 (d, J = 5.8 Hz, 2H), 8.83 (d, J = 6.4 Hz, 2H), 8.32 (s, 2H), 7.59 (d, J = 5.4 Hz, 2H), 4.40 (s, 3H).

[Fe-hbpy^{+}]; ^{1}H NMR (400 MHz, D_2O) δ 9.21 (d, *J* = 6.5 Hz, 2H), 8.91 (d, *J* = 6.4 Hz, 2H), 8.37 (d, *J* = 6.3 Hz, 2H), 7.62 (d, *J* = 6.9 Hz, 2H), 4.65 (t, *J* = 7.2 Hz, 2H), 2.05 (t, *J* = 7.3 Hz, 2H), 1.35 (m, 4H), 1.27 (m, 4H), 0.86 (t, *J* = 6.1 Hz, 3H).

[Fe-mpz^{+}]; ^{1}H NMR (400 MHz, D_2O) δ 9.70 (d, *J* = 4.4 Hz, 2H), 8.18 (s, 2H), 4.09 (s, 3H).

3. Experimental Procedures

3.1 Chemicals

Water for UV-Vis SEC and electrochemical measurements was de-ionized from an in-house supply and subsequently purged of oxygen by 3 freeze-pump-thaw cycles.

3.2 Material Characterization

Nuclear Magnetic Spectrum of molecules measured on Bruker Spectrospin Advance DPX 400 spectrometer. Chemical shifts were presented in parts per million (ppm). Fourier-transform infrared spectroscopy (FTIR) spectra were measured using a Bruker ALPHA Platinum-ATR spectrometer in the 4000–400 cm^{-1} range. Energy dispersive X-ray spectroscopy (EDS) analysis and Scanning electron microscopy (SEM) were performed by FEI - Quanta 200 FEG to characterize the morphology and depict the atomic ratios, respectively. UV-Vis analysis was performed by employing an Agilent Technologies Cary 300 UV–Vis spectrophotometer. XPS (Thermo Fisher Scientific; Al K-Alpha radiation; hυ = 1486.6 eV) measurement was also operated at survey mode by operating a flood gun for surface charge neutralization with 30 eV pass energy and 0.1 eV step size, and it

was performed for determining the elemental analysis. The correction of peak positions was calibrated by referencing the C1s peak position (284.8 eV) and shift other peaks in the spectrum accordingly. Elemental analyses were performed with a Thermo Scientific FLASH 2000 CHNS/O analyzer.

3.3 Electrochemistry

Electrochemistry experiments were conducted in a nitrogen-purged three neck round bottom flask, using a three-electrode setup of a 6 mm diameter glassy carbon disk working electrode, a Pt wire counter electrode and a leakless Ag/AgCl reference electrode. Measurements were conducted from a Zennium Pro (Zahner, Germany). Solutions of 0.1 mM sample and 0.1 M KCl were prepared inside a nitrogen purged glovebox. Standard measurement usually included a multi-cycle CV with a scan rate of 50 mV/s and a square wave voltammogram with a step height of 5 mV, pulse height of 100 mV, pulse width of 100 ms, and step width of 150 ms.

3.4 UV-Vis SEC

UV-Vis spectroelectrochemical (UV-Vis SEC) spectra were recorded inside a nitrogen glove box using an AvaSpec-ULS2048XL-EVO spectrometer (Avantes, Netherlands) with a AvaLight DH-S-BAL deuterium halogen light source (Avantes, Netherlands). Electrochemical experiments were carried out in a spectroelectrochemical cell (Hellma, Bioanalalytical Systems, USA) with a path length of 2 mm using a VersaSTAT 3 potentiostat (Princeton Applied Research, USA). The free hanging three electrode-setup consist of a glassy carbon working electrode, a platinum wire counter electrode and a Ag/AgCl wire reference electrode. For a typical UV-Vis SEC experiment, first a CV measurement of an aqueous solution of 0.1 mM analyte and 1 M KCl was conducted to confirm oxidation and reduction potentials for this electrode setup. After a short waiting time, to accommodate for potential product diffusion and equilibrate movement of free hanging electrodes (small drifts from vibrations inside the glovebox are possible and affect the baseline), a baseline was measured with the solution-electrode setup *as it is*. For the UV-Vis SEC measurement continuous UV-Vis spectra were measured each second and a stepwise increasing potential close to the respective oxidation potential was applied (potential change by 0.2-0.3 V each 2 minutes, e.g., for Fe oxidation at 0.4 V vs Ag/AgCl, 0.2 V, 0.5 V and 0.8 V were applied). After the experiment, the setup was cleaned, and fresh stock solution used for the reduction. For the reduction the stepwise decreasing potential directly was applied and continuous UV-Vis spectra, using a new baseline, were measured.
Data was analysed using OriginPro 2023 (OriginLab, USA). Spectra towards the last 20 s of relevant oxidation/reduction processes were summed up to get UV-Vis signatures of these reduction/oxidation processes.

Oxidation of the complexes **[Fe-hbpy$^+$]**, **[Fe-mbpy$^+$],** and **[Fe-mpz$^+$]** (Fe(II) -> Fe(III)) yields a loss of the respective MLCT transition and at 235 nm as the original chromophores degrade. **[Fe-mbpy$^+$]** and **[Fe-hbpy$^+$]** show a strong positive signal at 305 nm with a shoulder at 400 nm, while **[Fe-mpz$^+$]** shows positive signals at 300 nm and 420 nm. Reduction of the ligands of **[Fe-mbpy$^+$]** and **[Fe-hbpy$^+$]** show negative differential absorption at 284 nm, which is in line with the steady-state absorption and a strong positive differential absorption at 375 nm alongside a broad, weak positive differential absorption at 595 nm (**[Fe-mbpy$^+$]**), respective a very weak positive differential absorption at 570 nm (**[Fe-hbpy$^+$]**), which decays to a negative differential absorption mimicking the MLCT transition, when increasing negative potential. Similar signals can be observed when reducing the free ligands (**mbpy**, **hbpy**, see SI). Interestingly, one would expect a negative differential absorption at 530 nm as the chromophore of the MLCT is broken, while we observe only a positive differential absorption in this region, which are likely assigned to π*->π*-transitions on the bipyridinium moiety.[5] These π*->π*-transitions therefore must have a higher extinction coefficient in respect to the lost MLCT. When comparing the ligand spectra (**mbpy**) with the complex spectrum (**[Fe-mbpy$^+$]**), we see that the positive differential absorption at 595 nm is weaker compared to the positive differential absorption at 284 nm for the complex, indicating a superposition of a bleach of the MLCT and a gain, caused by the new π*->π*-transition. The UV-Vis spectrum upon reduction of **[Fe-mpz$^+$]** looks slightly different. Like the earlier discussed **[Fe-hbpy$^+$]** and **[Fe-mbpy$^+$]** can negative differential absorptions at 284 nm and a positive differential absorption at 365 nm be observed. An additional positive peak at wavelengths close to the MLCT is not observed. Reduction of **[Fe-mpz$^+$]** rather yields to a ground state bleach at 656 nm, which corresponds to the loss of MLCT.

3.5 fs-TA

Femtosecond transient absorption spectra were recorded on a custom experimental setup, which has been described previously.[6] A Ti:sapphire amplifier (Coherent, USA) with a pulse energy of 5 mJ, a repetition rate of 1 kHz, a laser wavelength of 800 nm and a pulse duration of 125 fs, is used as the fundamental to feed (i) a rotating CaF_2 window to generate a white light continuum from 300 nm to 800 nm (so called white light or probe beam) and (ii) an optimal parametrical amplifier (TOPAS prime, Light conversion, Lithuania) for generating tunable laser pulses in the UV to NIR range (so called pump beam).

The repetition rate of the pump beam is reduced to 0.5 kHz with a mechanical chopper. The polarisation of the pump beam is adjusted using a Berek compensator – polarizer combination and is set to 54.7° in respect to the polarisation angle of the white light. The pump beam is attenuated to 0.6 μJ/cm^2 and both beams are focused on a standard quartz cuvette or a quartz flow cuvette with 1 mm path length. The probe beam alongside a reference white light probe beam is coupled in a 150 mm focal length Czerny-Turner spectrograph (SP2150, Princeton Instruments, USA) with a two CCD array (Pascher Instruments, Sweden).
Data was chirp corrected, analysed and fitted using a python based script "KiMoPack".[7] Global analysis by assuming a 2-component decay exponential model was used for fitting. Due to the coherent artifact, were the first 400 fs (800 fs for the flow cell) removed from the fitting procedure. Spectra were edited using Origin (Originlab, USA).
To confirm photostability, UV-Vis spectra were conducted before and after TA measurement and additional TA measurements in a 1 mm flow cuvette were conducted with a flow rate of 0.1 mL/min (~0.1 cm/min at laser position), which yielded similar spectra as measurements in a normal 1 mm cuvette (see Figure S12).

3.6 Photodecomposition experiments

For illumination experiments, **[Fe-mpby$^+$]**, **[Fe-hpby$^+$]**, **[Fe-mpz$^+$]** and **[Fe-NH$_3$]** were dissolved in water to yield concentrations between 33 μM and 330 μM. Solutions were place in 1 cm cuvette in a Jasco V760 (Jasco Corporations, Japan). Samples were illuminated with Thorlabs LEDs (United States) over an area of 1 cm^2 over a certain amount of time (usually 10 to 30 min) with an active stirring bar to achieve mixing. After the set amount of time, the stirring bar was turned off and the solution was given time to equilibrate for 1 minute. Afterwards a standard UV-Vis spectrum was taken and the whole procedure repeated for up to 15 h.
Photodecomposition quantum yields were based on following citation.[8]
LEDs used: whitelight 4700 K MnWHL4 (22.5 mW/cm^2), 405 nm M405L3 (70 mW/cm^2), 505 nm M505L3 (19 mW/cm^2), 590 nm M590 L2 (2.5 mW/cm^2), 660 nm M660L4 (33.5 mW/cm^2).

3.7 Photocatalytic Experiments

The amount of photogenerated O_2 was recorded by gas chromatography (Agilent 7820A GC) equipped with a 5 Å molecular sieve column (Ar as the carrier gas) and a TCD detector. The photocatalytic experiments for oxygen evolution were performed in a 10 mL gas-tight Pyrex cell.
The dyad material (10 mg) and $Na_2S_2O_8$ (20 mM) sacrificial agent was dispersed in a 10 mL phosphate buffer solution (PBS, pH 7.1). The reaction mixture was degassed with N_2 gas for 30 min. before each experiment. The reaction flask was coupled to a solar light simulator (Sciencetech, Model SLB-300B, 300 W Xe lamp, AM 1.5 global filter) and calibrated to 1 sun (100 MW cm^{-2}). The above solution was also irradiated by visible light illumination (λ > 420 nm) through a 420 nm cut-off filter. The oxygen content in the headspace of the flask was sampled through the septum using a syringe and injected to GC 2 to 3 times each time. During the experiments, the mixture was magnetically stirred and no leakage from air in the reaction flask was determined by monitoring the N_2 content. Cycled OER experiments were performed by changing the solution after the powder is settled. The concentration of PBS and persulfate is the same in all cycles. Each data point is the mean of two measurements. The error margins in visible-light OER experiments are obtained from three different experiments. The margin is within 68% CI.

4. Computational Details

Quantum chemical simulations were performed using the Gaussian 16 package[9] in order to investigate structural and electronic properties of **[Fe-mpz⁺]** and **[Fe-mbpy⁺]**. Initially, the fully relaxed equilibrium structure of the singlet (S_0) and triplet (T_1) ground state structures were obtained at the density functional level of theory. In addition, the singlet ground state was relaxed at within the C_s point group. The B3LYP[10,11] exchange-correlation functional was applied in combination with the all-electron def2-SVP basis set. Implicit solvent effects (water: ε = 78.3553) were taken into account on the ground state properties by the solute electron density (SMD) variant of the integral equation formalism of the polarizable continuum model (equilibrium procedure).[12] All calculations were performed including D3 dispersion correction with Becke-Johnson damping.[13] Subsequently, a vibrational analysis was carried out for each optimized ground state structure (*i.e.*, S_0 and T_1) to verify that a minimum on the 3*N*-6-dimensional potential energy (hyper-)surface (PES) was obtained.

In the following, the Franck-Condon photophysical properties of both Fe(II) complexes were investigated by means of time-dependent DFT (TDDFT). To this aim, the 50 lowest singlet-singlet transitions as well as the 50 lowest spin-forbidden singlet-triplet transitions were obtained within the S_0 geometry using the same XC functional and basis set as for the preliminary ground state calculations. Thereby, the focus was set on the $^{1/3}$MLCT transitions form the iron to the aromatic ligand as well as on iron's $^{1/3}$MC (metal-centred) states. Such computational protocol allows a balanced description of locally excited states, *e.g.* MC and intra-ligand states, as well as of charge transfer states, *e.g.* MLCT, ligand-to-metal charge transfer (LMCT) and ligand-to-ligand charge transfer (LLCT) states, of 3d transition metal complexes as benchmarked with range-separated and meta-GGA functionals and multiconfigurational methods.[14,15] The non-equilibrium procedure of solvation was applied for the calculation of the excitation energies within the Franck-Condon. This procedure is well adapted for processes where only the fast reorganization of the electronic distribution of the solvent is important. Furthermore, we aimed to investigate the Franck-Condon photophysics of **[Fe-mpz⁺]** by means of multiconfigurational simulations in order to provide an elaborate description of both static (RASSCF) and dynamic (RASPT2) electron correction. Unfortunately, a stable closed-shell ground state wavefunction could only be obtained by incorporating all ten pairs of $\pi_{\mathrm{CN}}/\pi^*_{\mathrm{CN}}$ orbitals in addition to the five 3d and five 4d orbitals of the Fe(II), the two respective lone pairs, which form the coordinative σ bonds (interactive with $3d_{x^2-y^2}$ and $3d_{z^2}$), and the 3 pairs of $\pi_{\mathrm{mpz}}/\pi^*_{\mathrm{mpz}}$ orbitals. Despite the reduced computational demand of RASSCF with respect to the CASSCF methodology and making use of the molecular symmetry (C_S), such an active space of (36,38) proved to be computationally unfeasible.

Additionally, the excited state properties were evaluated within the relaxed triplet ground state structures of both complexes, which was predicted by DFT to be of ^{3}MLCT$_{\mathrm{mpz}}$ and ^{3}MLCT$_{\mathrm{mbpy}}$ character, respectively. Additionally, the lowest energy ^{3}MC state of **[Fe-mpz⁺]** (^{3}MC$_{\mathrm{mpz}}$) was (pre)relaxed by TDDFT (SMD with equilibrium procedure)[16] to T_1 and subsequently fully relaxed by DFT. Contributions of a ^{5}MC state to the photochemistry of **[Fe-mpz⁺]** were not investigated further as DFT-optimization of this state leads to the dissociation of the **mpz⁺** ligand.

Careful evaluation of the DFT and TDDFT results using merely the implicit SMD solvent model reveals that such model is insufficient to provide an unambiguous description of ground and excited state properties of the complexes, as shown exemplarily in Figure S13 for **[Fe-mpz⁺]**. Surprisingly, TDDFT predicts the ^{3}MLCT states (singlet-triplet transitions) well below the singlet ground state. Notably, the energy of the lowest and corresponding ^{3}MLCT state is approximately 2 eV higher as obtained by (unrestricted). Therefore, all previous simulations were also performed by incorporating explicit solvent effects, i.e. hydrogen bonds, as given by the first solvent shell in the vicinity of the $[Fe(CN)_5]^{3-}$ fragment. To this aim, the CREST package[17] was utilized. Ten H_2O molecules were found to be sufficient to coordinate to the $Fe(CN)_5]^{3-}$ fragment; less solvent molecules are insufficient while a larger number of water molecules decreases the average stabilization provided per solvent molecule. Explicit interactions with the coordinated **mpz⁺** and **mbpy⁺** were found to be neglectable. The combination of explicit and implicit solvent effects provides a consistent picture based on the DFT and TDDFT results. Thus, all ground and excited state simulations were rerun using this computational setup.

Furthermore, the 50 lowest spin and dipole-allowed triplet-triplet transitions were simulated at the TDDFT level of theory to model the excited state absorption as measured by means of transient absorption (TA) spectroscopy. These simulations were performed exclusively based on the combined explicit-implicit solvent model approach. TA spectra were predicted as difference spectra based on the excited state absorption stemming from triplet-triplet simulation (within ^{3}MLCT and ^{3}MC equilibria, T_1) and the singlet-singlet transitions within the Franck-Condon point. Thereby, a relative population of 1:1 was assumed. This procedure was applied previously and is capable to model the TA spectra at long delay times.[15,18–20]

Finally, the activation energy between the equilibrated ^{3}MLCT and ^{3}MC species was estimated at the unrestricted DFT level of theory. To this aim, the respective ^{3}MLCT and ^{3}MC equilibrium structures (including explicit and implicit solvent) were connected along linear-interpolated internal coordinates. Subsequently, the solvent environment of the highest energy image was fully relaxed, while the respective complex remained frozen. Noteworthy, the transition state structures obtained could not be fully relaxed to first-order saddle points and two imaginary normal modes remained. Unfortunately, all further attempts to fully relax these approximate transition state structures failed. This way, approximate activation energies of 0.20 and 0.22 eV were obtained for the ^{3}MLCT-^{3}MC pathway for **[Fe-mpz⁺]** and **[Fe-mbpy⁺]**. respectively.

All calculated equilibrium structures as well as high resolution images (charge density differences and spin densities) are available from the free online repository Zenodo.[21]

5. Results and Discussion

5.1 IR spectra

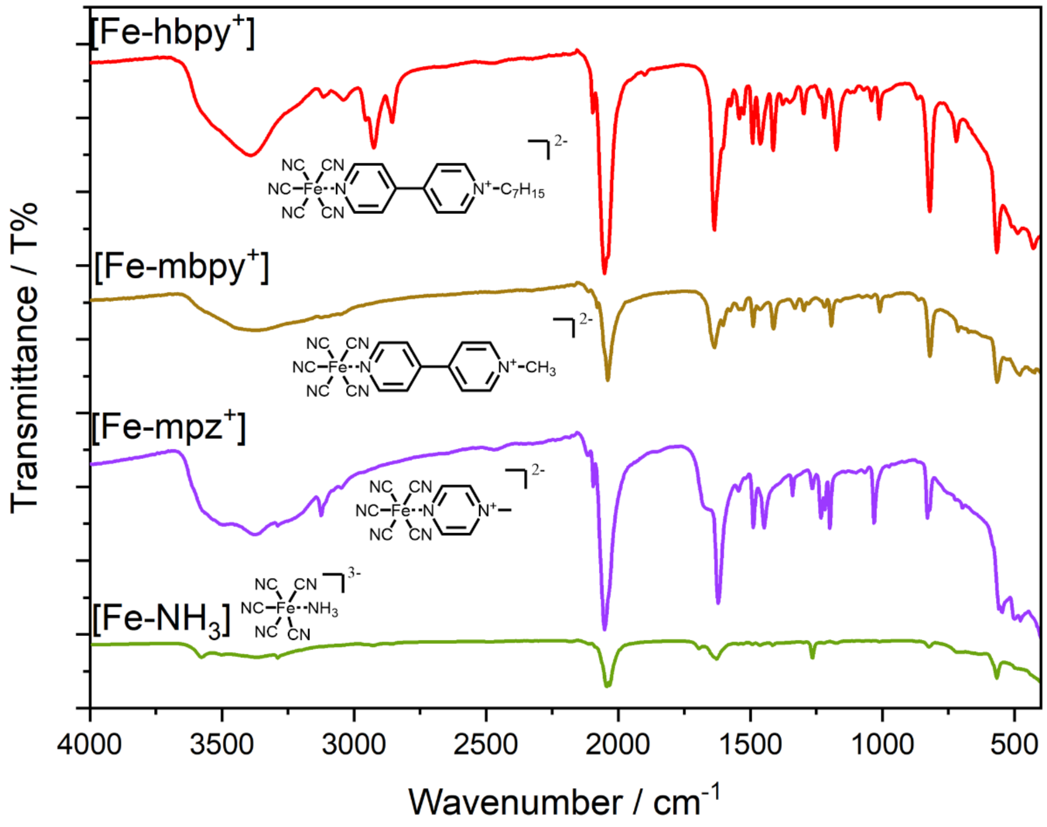

Figure S1. ATR-FTIR spectra of **[Fe-L⁺]** and **[Fe-NH$_3$]** complexes

5.2 NMR

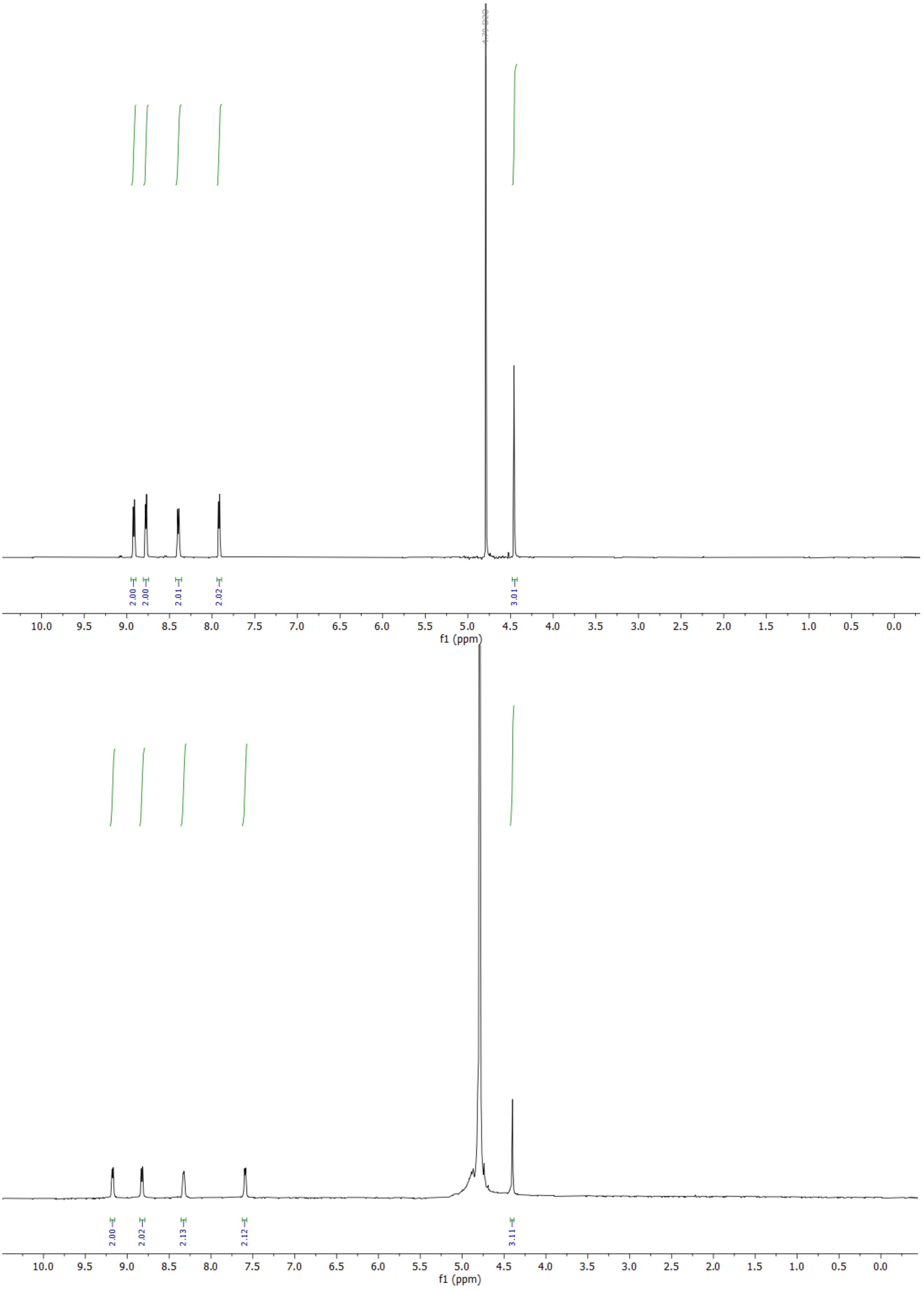

Figure S2. ^{1}H NMR spectra of mbpy^{+} (top) and **[Fe-mbpy^{+}]** (bottom). For mbpy^{+}; ^{1}H NMR (400 MHz, D_2O) δ 8.92 (d, J = 6.4 Hz, 2H), 8.78 (d, J = 6.4 Hz, 2H), 8.40 (d, J = 6.3 Hz, 2H), 7.92 (d, J = 6.4 Hz, 2H), 4.46 (s, 3H). For **[Fe-mbpy^{+}]**; ^{1}H NMR (400 MHz, D_2O) δ 9.18 (d, J = 5.8 Hz, 2H), 8.83 (d, J = 6.4 Hz, 2H), 8.32 (s, 2H), 7.59 (d, J = 5.4 Hz, 2H), 4.40 (s, 3H).

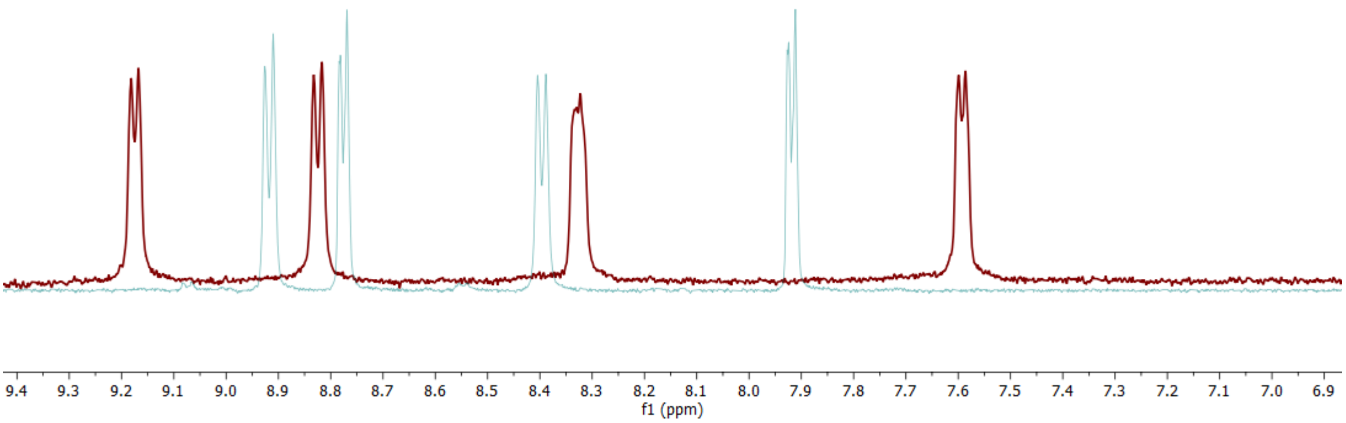

Figure S3. Overlay of ^{1}H NMR Spectra of **[Fe-mbpy⁺]** (maroon) and mbpy (teal), aromatic hydrogens only.

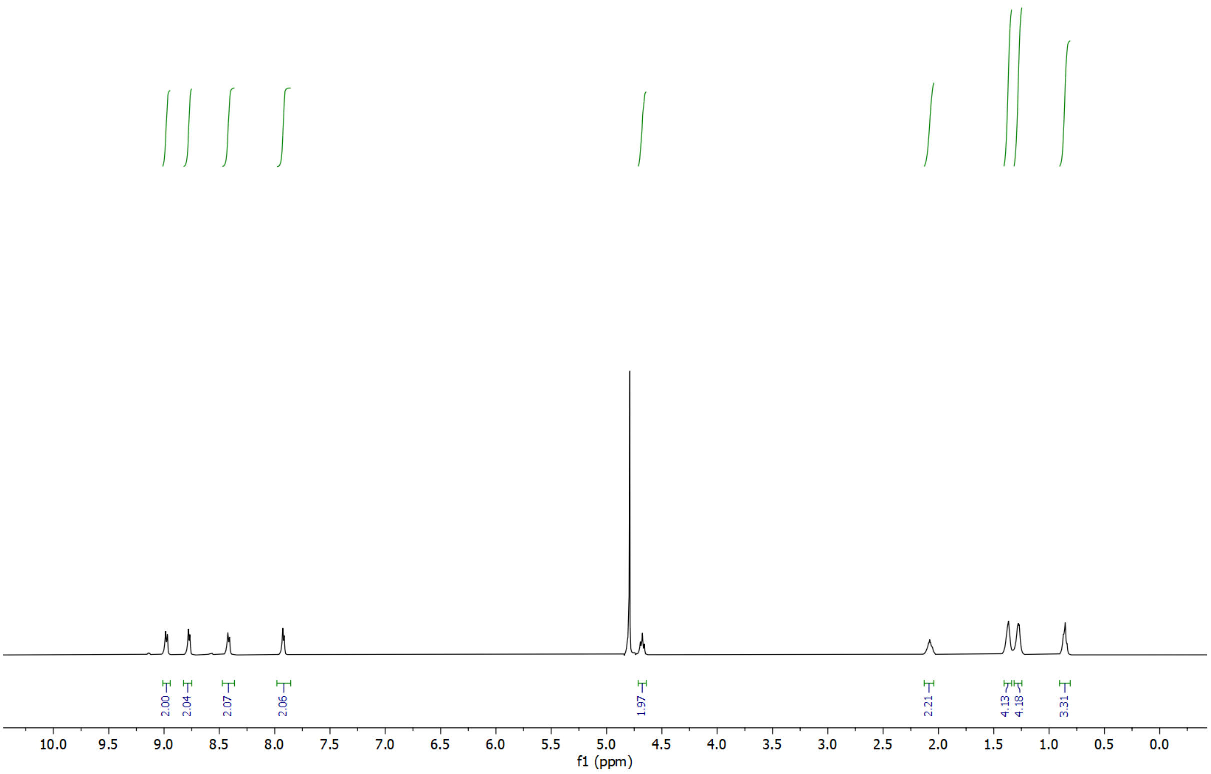

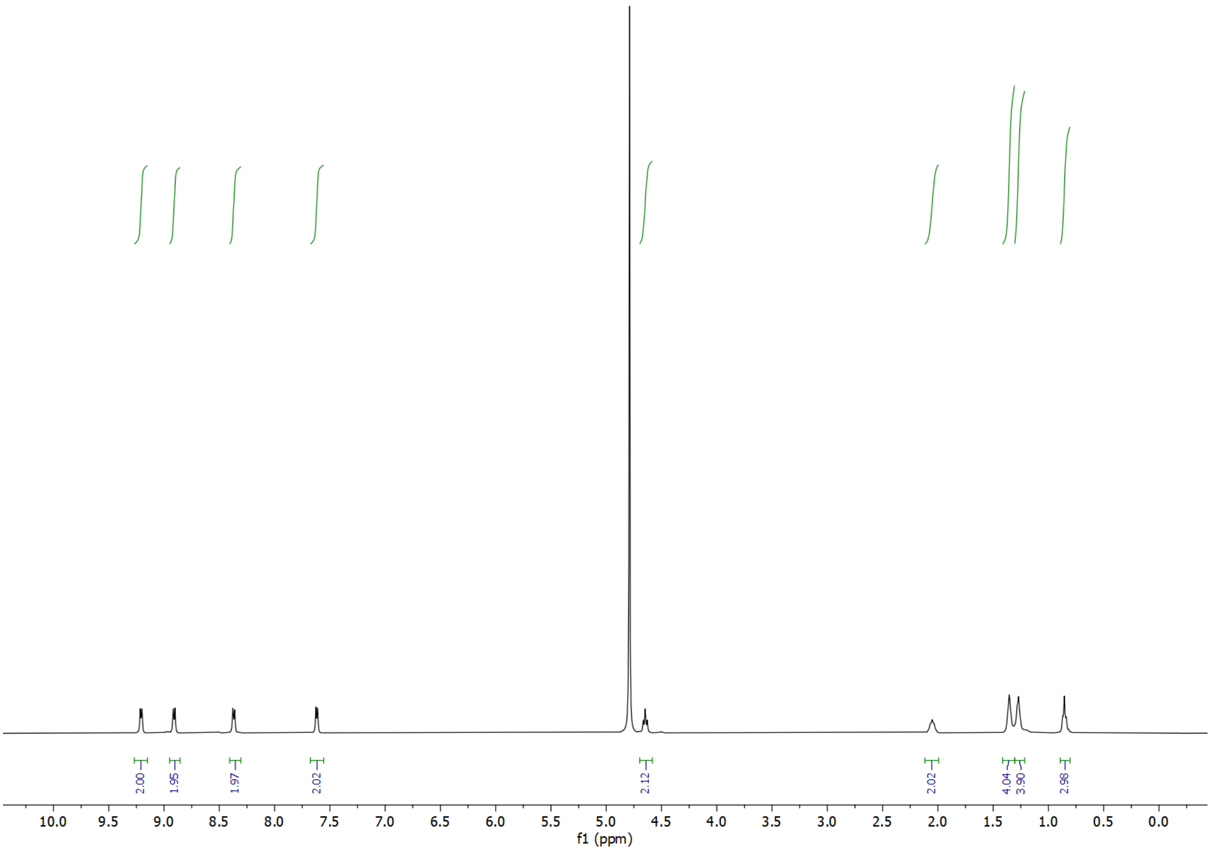

Figure S4. ^{1}H NMR spectra of hbpy^{+} (top) and **[Fe-hbpy^{+}]** (bottom). For hbpy^{+}; ^{1}H NMR (400 MHz, D_2O) δ 8.98 (d, *J* = 6.4 Hz, 2H), 8.79 (m, 2H), 8.42 (d, *J* = 6.1 Hz, 2H), 7.92 (m, 2H), 4.68 (t, *J* = 7.3 Hz, 2H), 2.08 (m, 2H), 1.37 (m, 4H), 1.28 (m, 4H), 0.85 (t, *J* = 6.9 Hz, 4H). For **[Fe-hbpy^{+}]**; ^{1}H NMR (400 MHz, D_2O) δ 9.21 (d, *J* = 6.5 Hz, 2H), 8.91 (d, *J* = 6.4 Hz, 2H), 8.37 (d, *J* = 6.3 Hz, 2H), 7.62 (d, *J* = 6.9 Hz, 2H), 4.65 (t, *J* = 7.2 Hz, 2H), 2.05 (t, *J* = 7.3 Hz, 2H), 1.35 (m, 4H), 1.27 (m, 4H), 0.86 (t, *J* = 6.1 Hz, 3H).

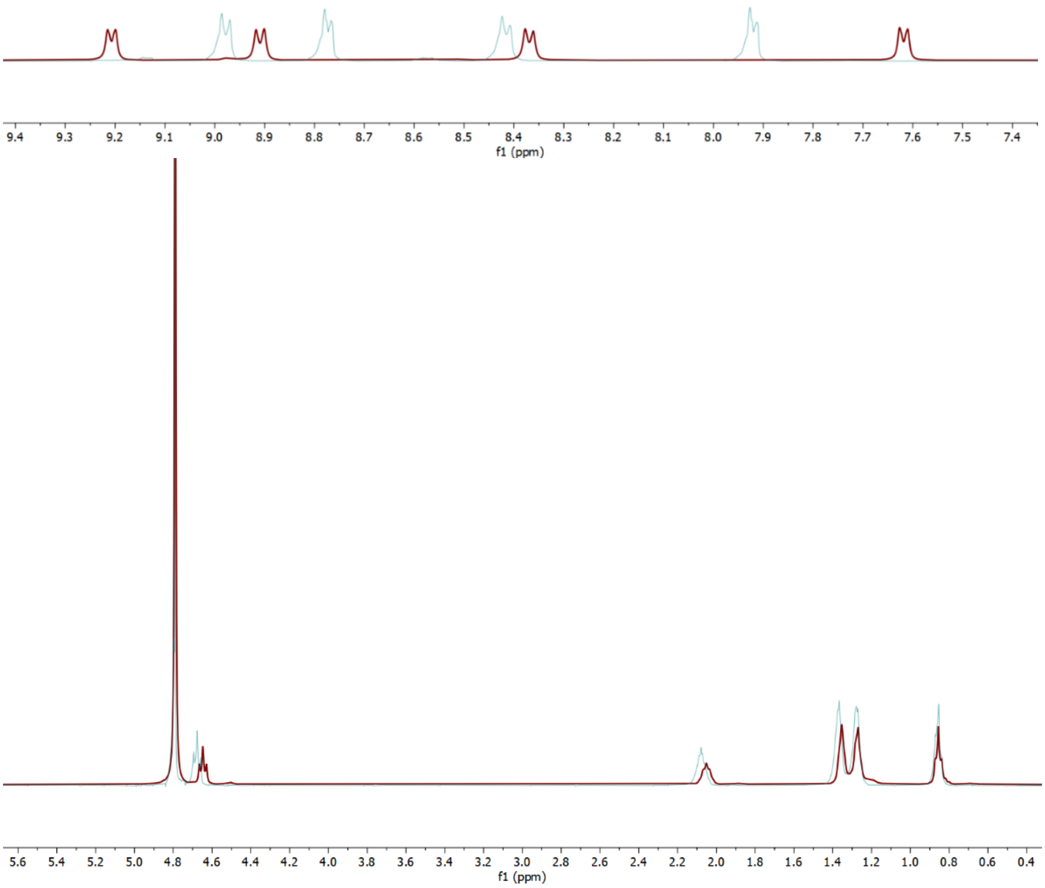

Figure S5. Overlay of ^{1}H NMR spectra of **[Fe-hbpy⁺]** (maroon) and hbpy⁺ (teal) for the aromatic (top) and aliphatic (bottom) region.

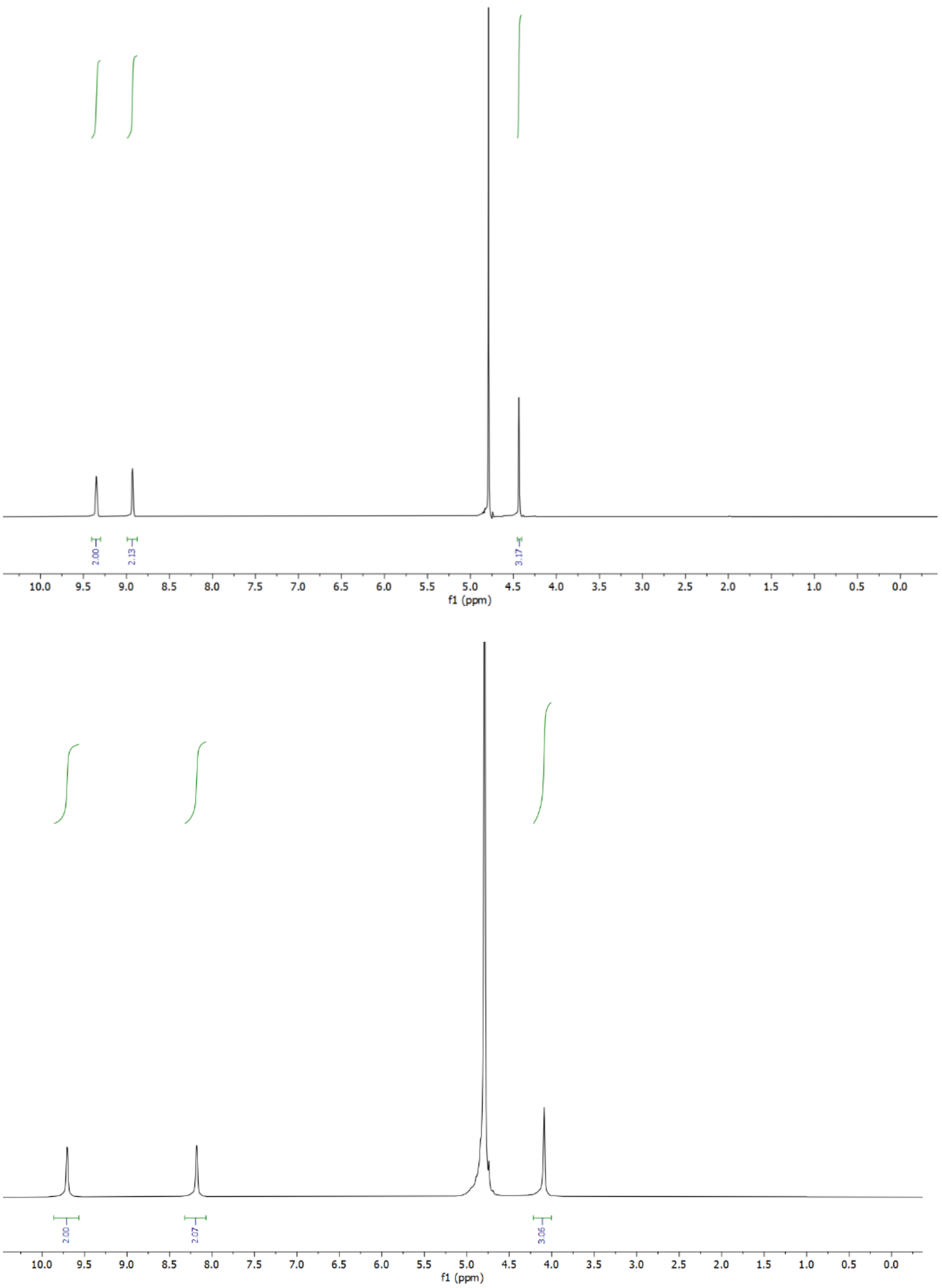

Figure S6. ^{1}H NMR spectra of N-methylpyrazinium iodide (mpz^{+}) (top) and **[Fe-mpz⁺]** (bottom). For mpz^{+}; ^{1}H NMR (400 MHz, D_2O) δ 9.35 (d, *J* = 2.7 Hz, 2H), 8.93 (d, *J* = 3.0 Hz, 2H), 4.44 (s, 3H). For **[Fe-mpz⁺]**; ^{1}H NMR (400 MHz, D_2O) δ 9.70 (d, *J* = 4.4 Hz, 2H), 8.18 (s, 2H), 4.09 (s, 3H).

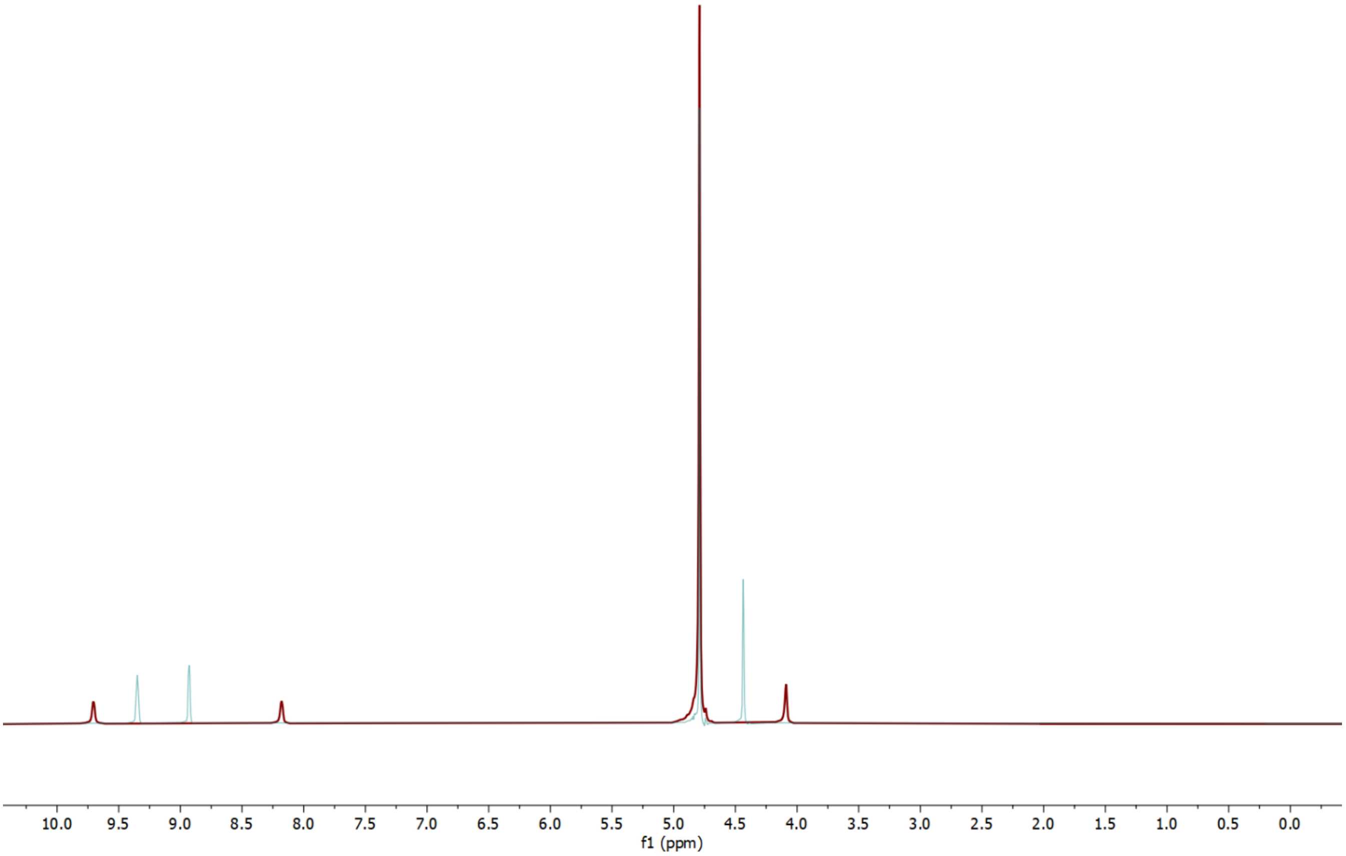

Figure S7. Overlay of ^{1}H NMR Spectra of **[Fe-mpz⁺]** (maroon) and mpz^{+} (teal).

5.3 Electrochemistry

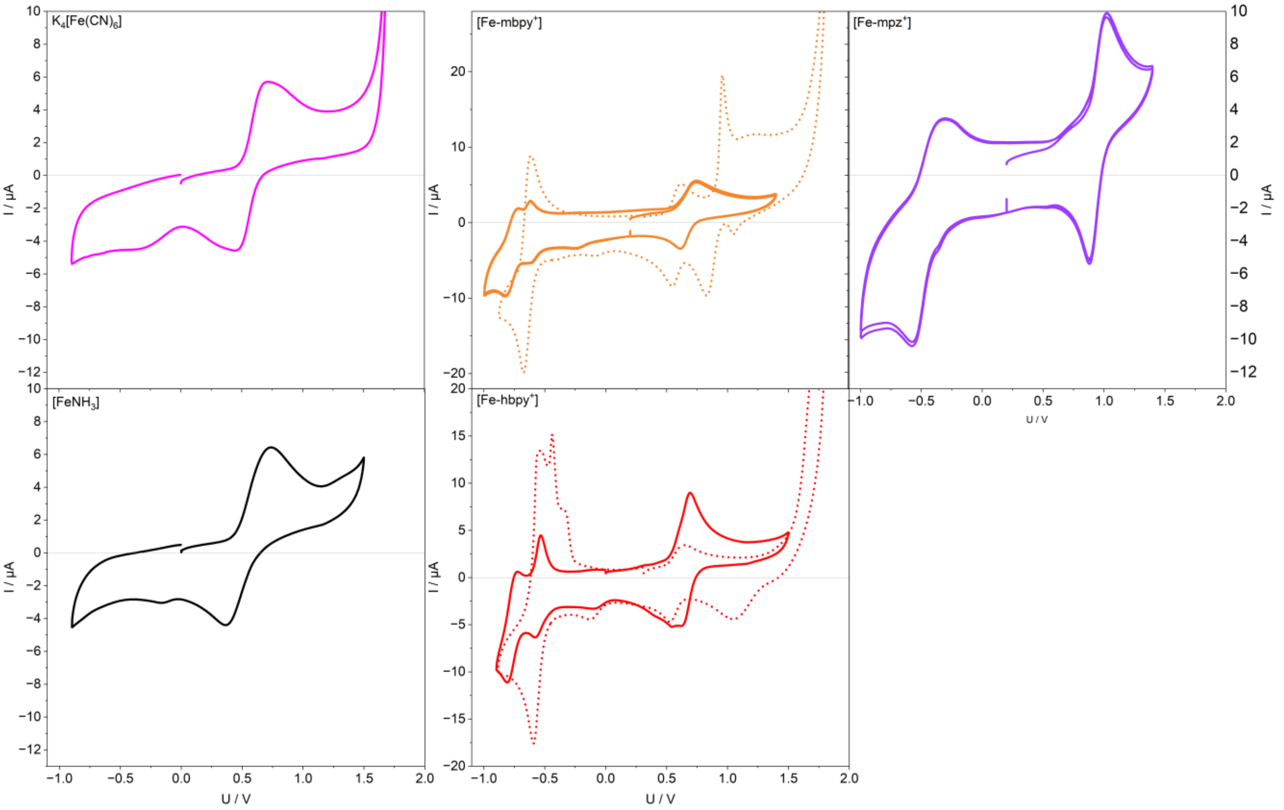

Figure S8. CV of aqueous solutions of Na$_4$[Fe(CN)$_6$], **[FeNH$_3$]**, **[Fe-mbpy$^+$]**, **[Fe-hbpy$^+$]** and **[Fe-mpz$^+$]** (full lines) and the ligands **mbpy** and **hpby** (dotted lines), 100 mV/s scan speed (Na$_4$[Fe(CN)$_6$] was added to **[Fe-mbpy$^+$]** and **[Fe-hbpy$^+$]**)

5.4 Transient absorption

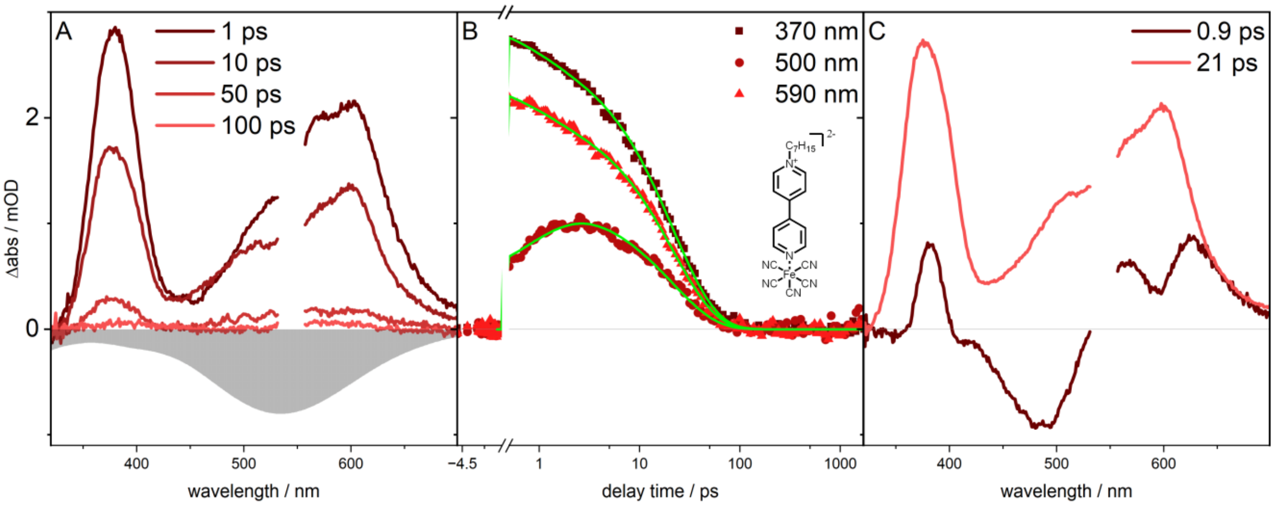

Figure S9. TA spectrum of an aqueous solution of **[Fe-hbpy⁺]** at selected delay times (lines) and scaled, inverted ground state absorption spectrum (grey area) (A), kinetic traces (dots) and their global fit traces (green lines) at selected wavelengths (B) and DAS (C).

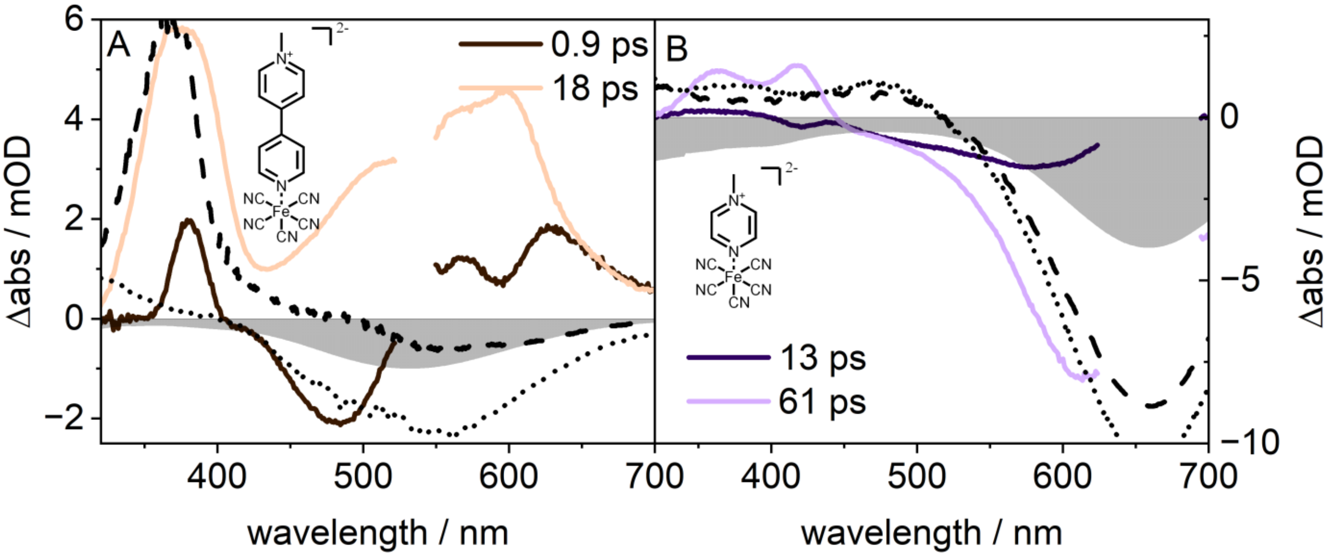

Figure S10. Decay associated spectra of **[Fe-mbpy⁺]** (A) and **[Fe-mpz⁺]** (B). Scaled UV-Vis SEC spectra (reduction: dotted line, oxidation: dashed line) and inverted ground state absorption spectra added for comparison.

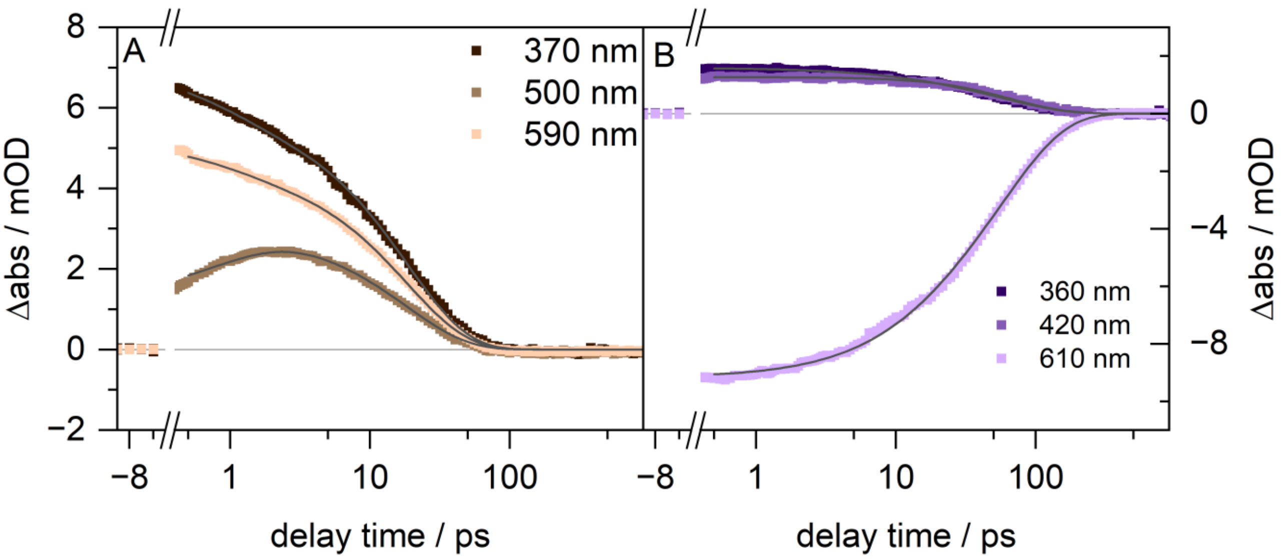

Figure S11. Kinetic TA traces of aqueous solutions of **[Fe-mbpy⁺]** (A) and **[Fe-mpz⁺]** (B) at selected wavelengths, excited at their respective MLCT transition (530 nm for **[Fe-mbpy⁺]**, 656 nm for **[Fe-mpz⁺]**). Black lines show the fit according to global analysis.

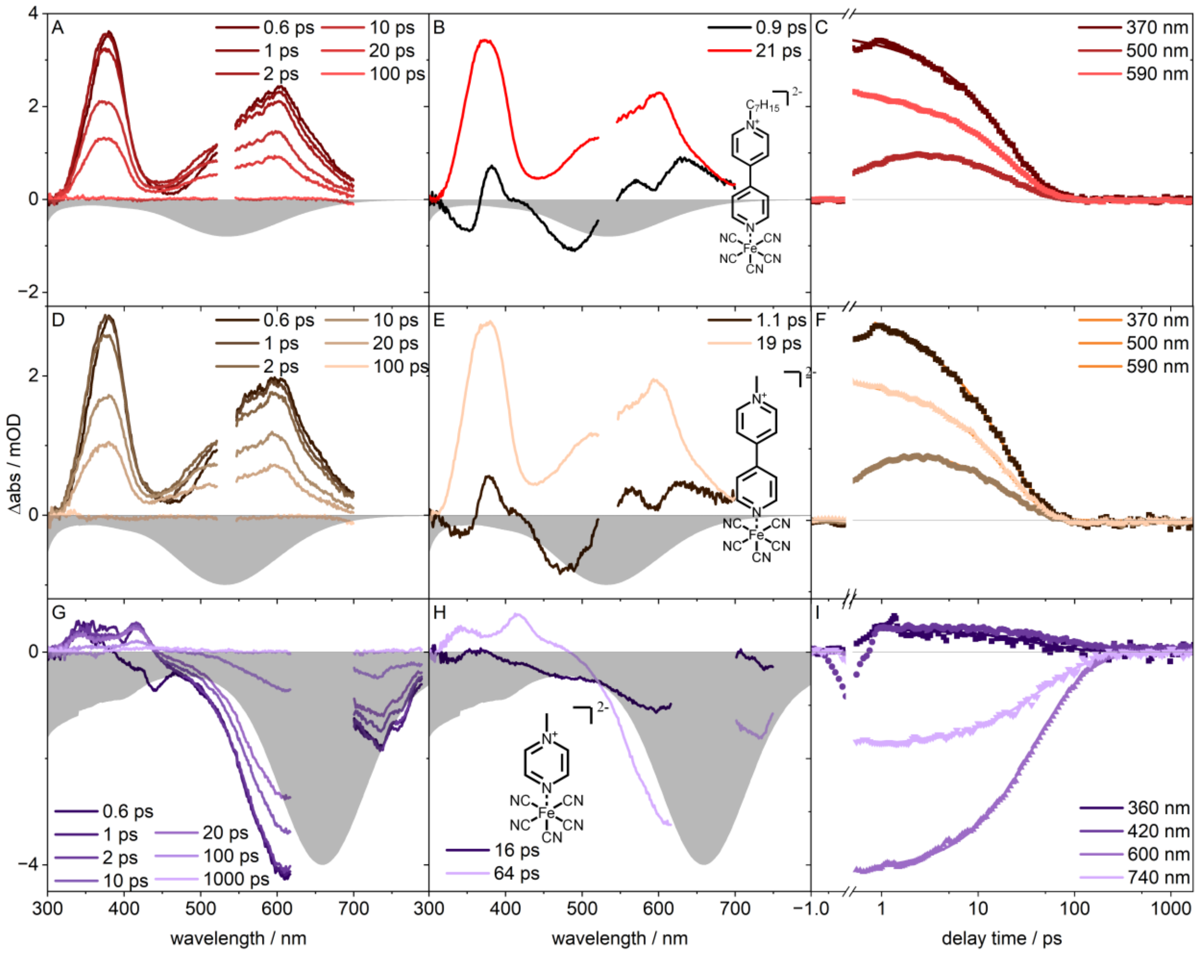

Figure S12. TA spectrum of an aqueous solution of **[Fe-hbpy⁺]** (A-C), **[Fe-mbpy⁺]** (D-F) and **[Fe-mpz⁺]** (G-I) at selected delay times (lines) and scaled, inverted ground state absorption spectrum (grey area) (A,D,G), DAS (B,E,H) and kinetic traces (dots) and their global fit traces (lines) at selected wavelengths (C,F,I). Measurements conducted in a flow cell with a flow rate of 0.1 ml/min (~0.1 mm/min).

5.5 Quantum chemical results

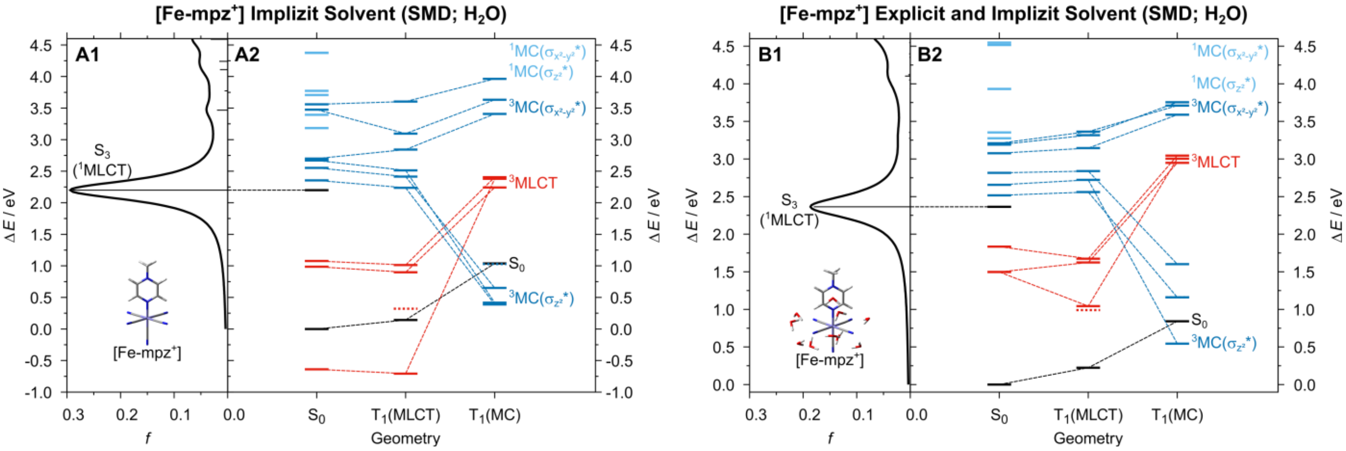

Figure S13. Simulated electronic absorption spectrum of **[Fe-mpz⁺]** as obtained at the TDDFT level of theory (B3LYP/def2-SVP), A1, in implicit water environment (SMD) and, B1, in a combined explicit-implicit water environment (10 water molecules + SMD). B1 and B2, excited state relaxation scheme involving the ground and excited state landscape within the fully relaxed S_0, T_1(^{3}MLCT) and T_1(^{3}MC) geometries (from left to right). Singlet states are shown in black, ^{3}MLCT states in red, ^{1}MC and ^{3}MC states are visualized in light and dark blue, respectively. Unrestricted DFT energies of ^{3}MLCT and ^{3}MC states are displayed in dashed.

Table S1. Simulated excited state properties such as electronic character, excitation energy, wavelength and oscillator strength within the S_0 equilibrium structure (C_1 with 10 H_2O molecules) related to prominent dipole-allowed singlet-singlet transitions contributing to the UV-Vis spectrum of **[Fe-mpz⁺]** as well as dipole-forbidden singlet-triplet transitions as obtained at the TDDFT level of theory (B3LYP/def2-SVP). Solvent (water) effects were considered by a combination of explicit (10 H_2O molecules) and implicit solvent effects (SMD).

Singlet-singlet transitions					Singlet-triplet transitions				
Excitation, $S_0 \rightarrow S_i$	Character	ΔE_{0i} / eV	λ / nm	f_{0i}	Excitation, $S_0 \rightarrow T_i$	Character	ΔE_{0i} / eV	λ / nm	f_{0i}
S_3	^{1}MLCT$_{mpz}$	2.36	524	0.1803	T_1	^{3}MLCT$_{mpz}$	1.50	828	0.0000
S_4	^{1}MC$_{z^2}$	3.28	379	0.0023	T_2	^{3}MLCT$_{mpz}$	1.80	689	0.0000
S_5	^{1}MC$_{z^2}$	3.35	370	0.0024	T_3	^{3}MLCT$_{mpz}$	1.84	676	0.0000
S_9	^{1}MC$_{z^2}$	3.93	315	0.0012	T_4	^{3}MC$_{z^2}$	2.52	493	0.0000
S_{10}	^{1}MC$_{x^2\text{-}y^2}$	4.04	307	0.0001	T_5	^{3}MC$_{z^2}$	2.66	467	0.0000
S_{13}	^{1}LLCT	4.10	302	0.0097	T_6	^{3}MC$_{z^2}$	2.82	440	0.0000
S_{20}	^{1}MC$_{x^2\text{-}y^2}$	4.52	275	0.0004	T_7	^{3}MC$_{x^2\text{-}y^2}$	3.08	403	0.0000
S_{21}	^{1}MC$_{x^2\text{-}y^2}$	4.55	273	0.0019	T_8	^{3}MC$_{x^2\text{-}y^2}$	3.19	389	0.0000
S_{32}	^{1}IL/^{1}LLCT	5.11	243	0.0304	T_9	^{3}MC$_{x^2\text{-}y^2}$	3.21	386	0.0000
S_{33}	^{1}LLCT	5.24	237	0.0237					
S_{46}	^{1}MC$_{\pi^*}$	5.68	218	0.0309					

Table S2. Electronic characters as visualized by charge density differences (CDDs) for singlet-singlet and singlet-triplet transitions obtained of **[Fe-mpz⁺]** as obtained at the TDDFT level of theory (B3LYP/def2-SVP). Solvent (water) effects were considered by a combination of explicit (10 H_2O molecules) and implicit solvent effects (SMD). Charge transfer takes place from red to blue.

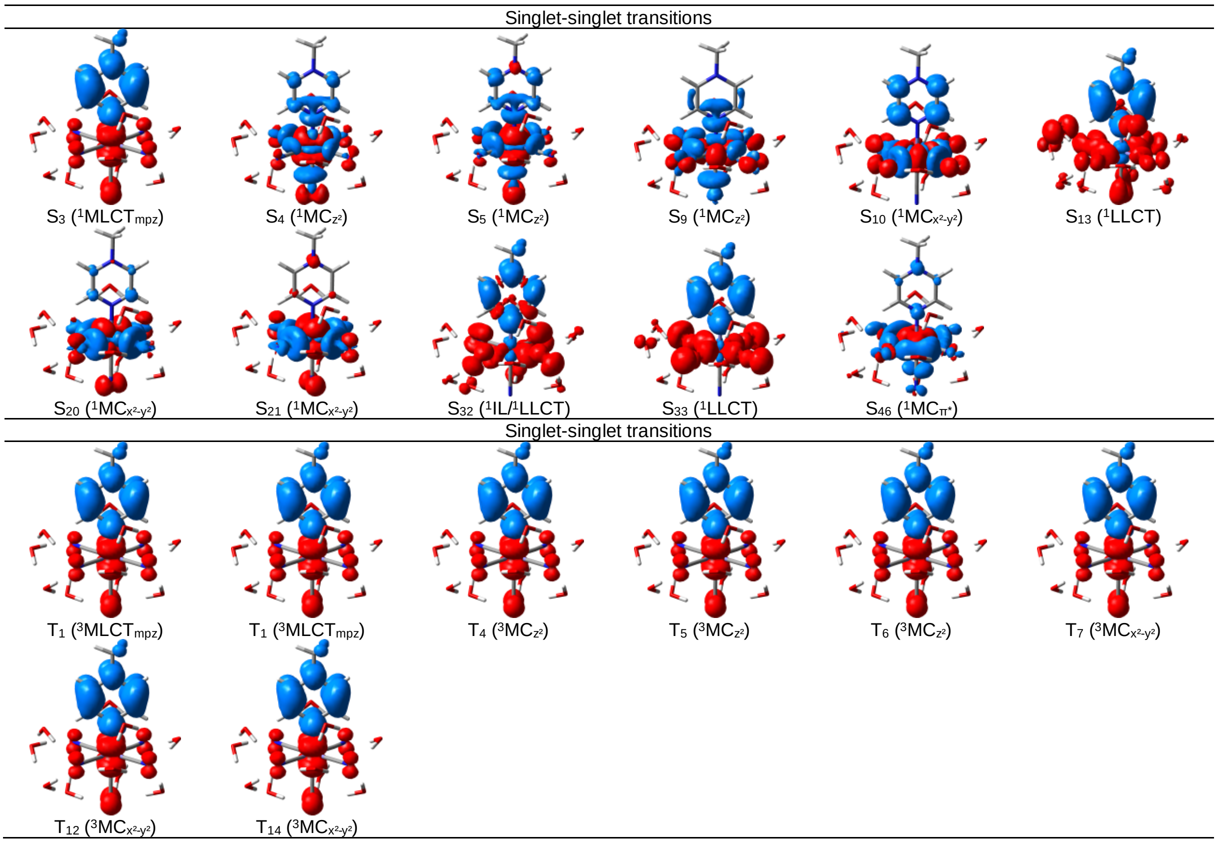

Table S3. Simulated excited state properties such as electronic character, excitation energy, wavelength and oscillator strength within the S_0 equilibrium structure (C_1 with 10 H_2O molecules) related to prominent dipole-allowed singlet-singlet transitions contributing to the UV-Vis spectrum of **[Fe-mbpy⁺]** as well as dipole-forbidden singlet-triplet transitions as obtained at the TDDFT level of theory (B3LYP/def2-SVP). Solvent (water) effects were considered by a combination of explicit (10 H_2O molecules) and implicit solvent effects (SMD).

Singlet-singlet transitions					Singlet-triplet transitions				
Excitation, $S_0 \rightarrow S_i$	Character	ΔE_{0i} / eV	λ / nm	f_{0i}	Excitation, $S_0 \rightarrow T_i$	Character	ΔE_{0i} / eV	λ / nm	f_{0i}
S_3	^{1}MLCT$_{mbpy}$	2.37	523	0.1613	T_1	^{3}MLCT$_{mbpy}$	2.10	590	0.0000
S_4	^{1}MC$_{z^2}$	3.24	383	0.0027	T_2	^{3}MLCT$_{mbpy}$	2.26	548	0.0000
S_5	^{1}MC$_{z^2}$	3.24	383	0.0029	T_3	^{3}MLCT$_{mbpy}$	2.29	542	0.0000
S_9	^{1}MC$_{z^2}$	3.86	321	0.0000	T_4	^{3}MC$_{z^2}$	2.47	502	0.0000
S_{17}	^{1}IL/^{1}LLCT	4.30	288	0.2411	T_5	^{3}MC$_{z^2}$	2.50	497	0.0000
S_{18}	^{1}MC$_{x^2-y^2}$	4.34	286	0.0003	T_6	^{3}MC$_{z^2}$	2.81	441	0.0000
S_{22}	^{1}MC$_{x^2-y^2}$	4.53	274	0.0003	T_7	^{3}MC$_{x^2-y^2}$	3.18	390	0.0000
S_{24}	^{1}MC$_{x^2-y^2}$	4.56	272	0.0016	T_8	^{3}MC$_{x^2-y^2}$	3.26	380	0.0000
S_{26}	^{1}IL/^{1}LLCT	4.63	268	0.1706	T_{10}	^{3}MC$_{x^2-y^2}$	3.29	350	0.0000
S_{27}	^{1}LLCT	4.69	264	0.0739					
S_{29}	^{1}LLCT	4.72	262	0.1055					

Table S4. Electronic characters as visualized by charge density differences (CDDs) for singlet-singlet and singlet-triplet transitions obtained of **[Fe-mbpy⁺]** as obtained at the TDDFT level of theory (B3LYP/def2-SVP). Solvent (water) effects were considered by a combination of explicit (10 H_2O molecules) and implicit solvent effects (SMD). Charge transfer takes place from red to blue.

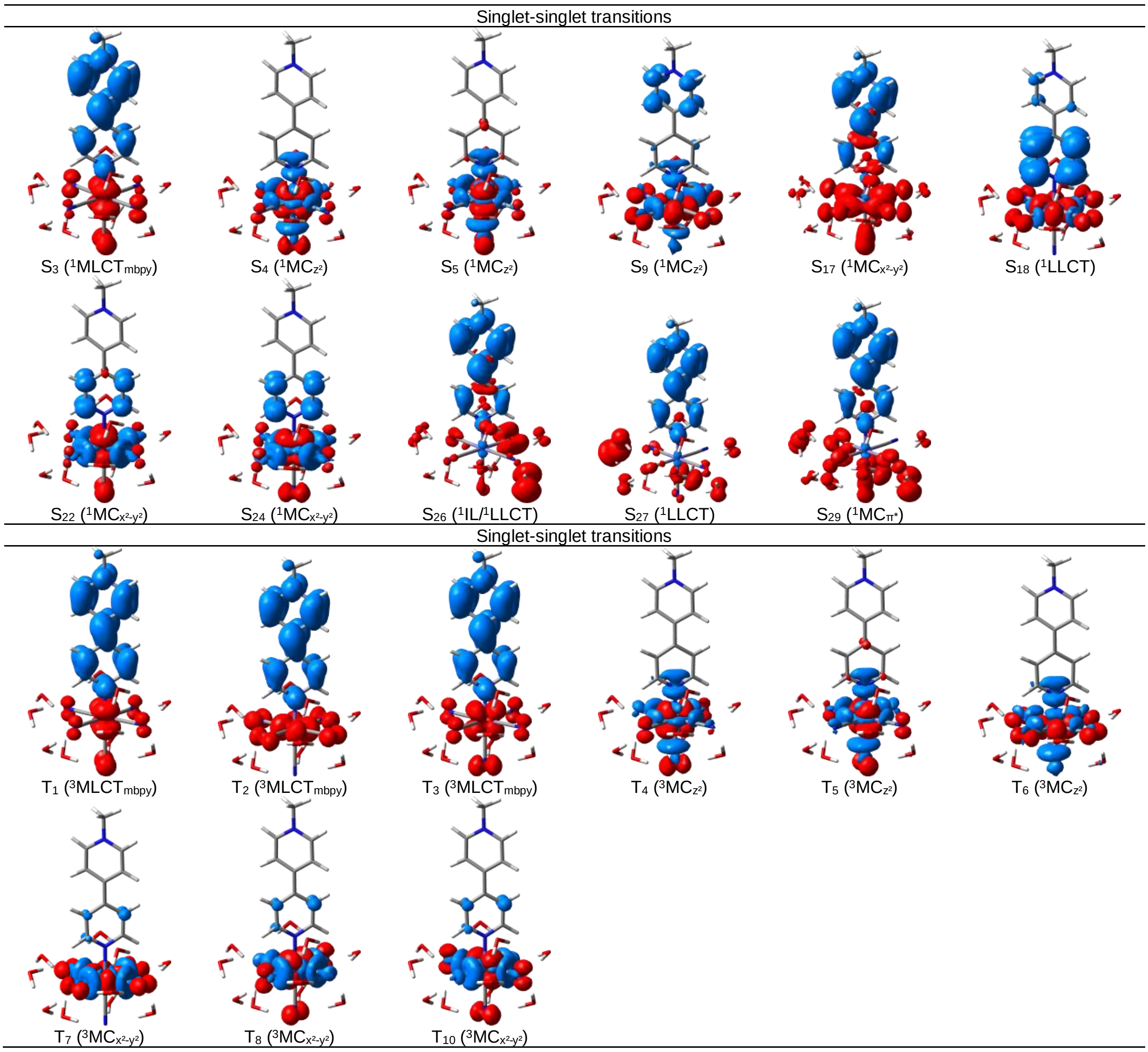

Table S5. Simulated excited state properties such as electronic character, excitation energy, wavelength, oscillator strength and spin contamination within the T_1 equilibrium structures or ^{3}MLCT (left) and ^{3}MC (right) character (C_1 with 10 H_2O molecules) related to prominent spin and dipole-allowed triplet-triplet transitions contributing to the excited-state absorption in the transient absorption spectrum of **[Fe-mpz⁺]** as obtained at the TDDFT level of theory (unrestricted B3LYP/def2-SVP). Solvent (water) effects were considered by a combination of explicit (10 H_2O molecules) and implicit solvent effects (SMD).

^{3}MLCT (T_1) equilibrium structure, triplet-triplet transitions						^{3}MC (T_1) equilibrium structure, triplet-triplet transitions					
Excitation, $T_1 \rightarrow T_i$	Character	ΔE_{1i} / eV	λ / nm	f_{1i}	$\langle s^2 \rangle$	Excitation, $T_1 \rightarrow T_i$	Character	ΔE_{1i} / eV	λ / nm	f_{1i}	$\langle s^2 \rangle$
T_{11}	$^3LMCT_{mpz}$	3.33	373	0.0478	2.09	T_4	$^3MLCT_{mpz}$	1.46	849	0.0176	2.27
T_{35}	$^3LMCT_{CN}$	4.30	289	0.0262	2.22	T_5	$^3MLCT_{mpz}$	1.56	796	0.0097	2.27
T_{36}	^{3}LLCT	4.30	288	0.0212	2.56	T_7	$^3MLCT_{mpz}$	1.95	636	0.0081	2.82
T_{41}	$^3LMCT_{CN}$	4.45	279	0.0202	2.15	T_{60}	$^3MLCT_{mpz}$	4.96	250	0.0218	2.37
T_{46}	^{3}IL	4.71	263	0.0454	2.05	T_{64}	$^3MLCT_{mpz}$	5.00	248	0.0080	2.49
T_{61}	$^3MC_{\pi}$	5.57	223	0.0276	3.34	T_{70}	$^3MLCT_{mpz}$	5.01	248	0.0187	2.63
T_{65}	$^3MC_{\pi}$	5.67	219	0.0337	3.50	T_{76}	$^3MLCT_{mpz}$	5.20	238	0.0333	2.86
T_{67}	^{3}LLCT	5.72	217	0.0409	2.26						
T_{72}	^{3}IMCT	5.79	214	0.0338	2.18						

Table S6. Electronic characters as visualized by charge density differences (CDDs) for singlet-singlet and singlet-triplet transitions obtained of **[Fe-mpz⁺]** as obtained at the TDDFT level of theory (B3LYP/def2-SVP). Solvent (water) effects were considered by a combination of explicit (10 H_2O molecules) and implicit solvent effects (SMD). Charge transfer takes place from red to blue; spin densities are provided for triplet ground states (T_1).

^{3}MLCT (T_1) equilibrium structure, triplet-triplet transitions

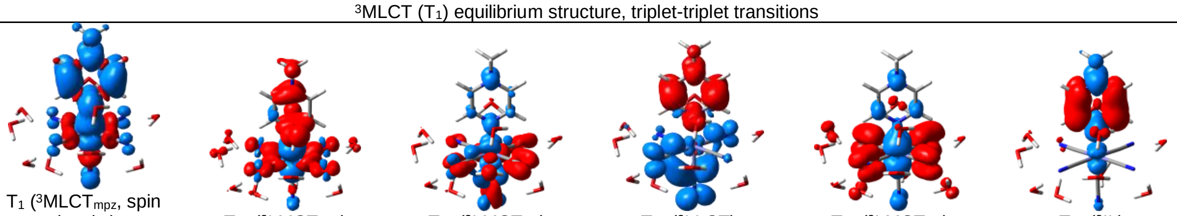

T_1 ($^3MLCT_{mpz}$, spin density) | T_{11} ($^3LMCT_{mpz}$) | T_{35} ($^3LMCT_{CN}$) | T_{36} (^{3}LLCT) | T_{41} ($^3LMCT_{CN}$) | T_{46} (^{3}IL)

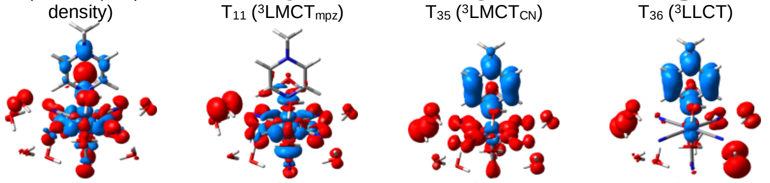

T_{61} ($^3MC_{\pi}$) | T_{65} ($^3MC_{\pi}$) | T_{67} (^{3}LLCT) | T_{72} (^{3}IMCT)

^{3}MC (T_1) equilibrium structure, triplet-triplet transitions

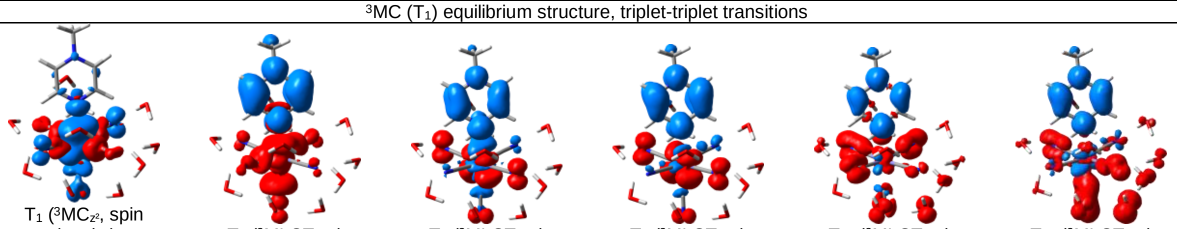

T_1 ($^3MC_{z^2}$, spin density) | T_4 ($^3MLCT_{mpz}$) | T_5 ($^3MLCT_{mpz}$) | T_7 ($^3MLCT_{mpz}$) | T_{60} ($^3MLCT_{mpz}$) | T_{64} ($^3MLCT_{mpz}$)

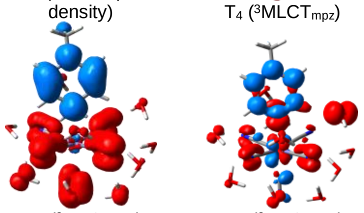

T_{70} ($^3MLCT_{mpz}$) | T_{76} ($^3MLCT_{mpz}$)

Table S7. Simulated excited state properties such as electronic character, excitation energy, wavelength, oscillator strength and spin contamination within the T_1 equilibrium structures or ^{3}MLCT (left) and ^{3}MC (right) character (C_1 with 10 H_2O molecules) related to prominent spin and dipole-allowed triplet-triplet transitions contributing to the excited-state absorption in the transient absorption spectrum of **[Fe-mbpy$^+$]** as obtained at the TDDFT level of theory (unrestricted B3LYP/def2-SVP). Solvent (water) effects were considered by a combination of explicit (10 H_2O molecules) and implicit solvent effects (SMD).

^{3}MLCT (T_1) equilibrium structure, triplet-triplet transitions						^{3}MC (T_1) equilibrium structure, triplet-triplet transitions					
Excitation, $T_1 \rightarrow T_i$	Character	ΔE_{1i} / eV	λ / nm	f_{1i}	$\langle s^2 \rangle$	Excitation, $T_1 \rightarrow T_i$	Character	ΔE_{1i} / eV	λ / nm	f_{1i}	$\langle s^2 \rangle$
T_7	^{3}IL	2.40	517	0.2121	2.04	T_6	$^3MLCT_{mbpy}$	1.95	634	0.0100	3.04
T_9	$^3LMCT_{mbpy}$	2.71	458	0.4607	2.15	T_{34}	$^3MLCT_{mbpy}$	4.39	283	0.4385	2.19
T_{21}	^{3}IMCT/^{3}IL	3.75	331	0.0762	2.13	T_{44}	$^3MLCT_{mbpy}$	4.61	269	0.1145	2.11
T_{22}	$^3LMCT_{mbpy}$	3.75	330	0.0553	2.07						
T_{23}	$^3LMCT_{mbpy}$	3.75	330	0.2631	2.22						

Table S8. Electronic characters as visualized by charge density differences (CDDs) for singlet-singlet and singlet-triplet transitions obtained of **[Fe-mbpy$^+$]** as obtained at the TDDFT level of theory (B3LYP/def2-SVP). Solvent (water) effects were considered by a combination of explicit (10 H_2O molecules) and implicit solvent effects (SMD). Charge transfer takes place from red to blue; spin densities are provided for triplet ground states (T_1).

^{3}MLCT (T_1) equilibrium structure, triplet-triplet transitions

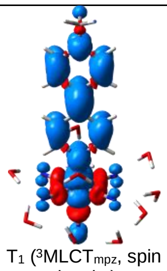	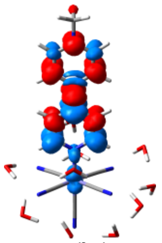	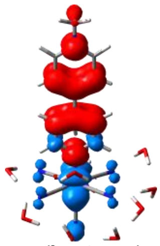	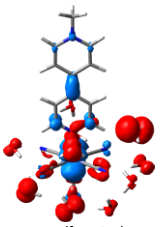	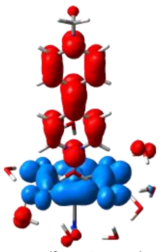	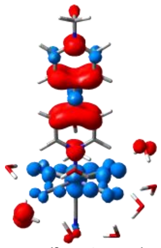
T_1 ($^3MLCT_{mpz}$, spin density)	T_7 (^{3}IL)	T_9 ($^3LMCT_{mbpy}$)	T_{21} (^{3}IMCT)	T_{22} ($^3LMCT_{mbpy}$)	T_{23} ($^3LMCT_{mbpy}$)

^{3}MC (T_1) equilibrium structure, triplet-triplet transitions

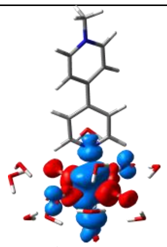	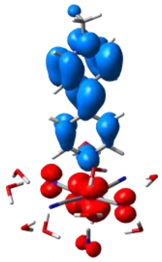	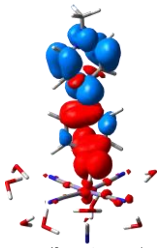	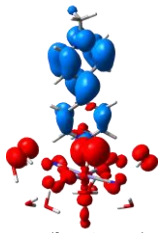
T_1 ($^3MC_{z^2}$, spin density)	T_6 ($^3MLCT_{mbpy}$)	T_{34} ($^3MLCT_{mbpy}$)	T_{44} ($^3MLCT_{mbpy}$)

Table S9. Simulated excited state properties such as electronic character, excitation energy, wavelength and oscillator strength within the S_0 equilibrium structure (C_s symmetry) related to prominent dipole-allowed singlet-singlet (top) transitions contributing to the UV-Vis spectrum of **[Fe-mpz⁺]** as well as dipole-forbidden singlet-triplet transitions as obtained at the TDDFT level of theory (B3LYP/def2-SVP). Solvent (water) effects were considered by a polarizable continuum model (SMD).

Singlet-singlet transitions					Singlet-triplet transitions				
Excitation, $S_0 \rightarrow S_i$ (irrep.)	Character	ΔE_{0i} / eV	λ / nm	f_{0i}	Excitation, $S_0 \rightarrow T_i$ (irrep.)	Character	ΔE_{0i} / eV	λ / nm	f_{0i}
S_3 (A’)	$^1MLCT_{mpz}$	2.20	564	0.2895	T_1 (A’)	$^3MLCT_{mpz}$	-0.65	-1939	0.0000
S_7 (A”)	$^1MC_{z^2}$	3.18	389	0.0007	T_2 (A’)	$^3MLCT_{mpz}$	0.99	1256	0.0000
S_8 (A’)	$^1MC_{z^2}$	3.39	365	0.0001	T_3 (A”)	$^3MLCT_{mpz}$	1.08	1151	0.0000
S_{11} (A”)	$^1MC_{x^2-y^2}$	3.71	334	0.0000	T_4 (A”)	$^3MC_{z^2}$	2.35	527	0.0000
S_{12} (A’)	$^1MC_{z^2}$	3.77	329	0.0057	T_5 (A’)	$^3MC_{z^2}$	2.55	486	0.0000
S_{18} (A’)	$^1MC_{x^2-y^2}$	4.37	283	0.0002	T_6 (A’)	$^3MC_{z^2}$	2.67	464	0.0000
S_{19} (A”)	$^1MC_{x^2-y^2}$	4.38	283	0.0003	T_7 (A’)	$^3MC_{x^2-y^2}$	2.70	459	0.0000
S_{29} (A”)	$^1IL/^1LLCT$	5.14	241	0.0763	T_{13} (A’)	$^3MC_{x^2-y^2}$	3.48	357	0.0000
S_{39} (A’)	^1LLCT	5.78	214	0.1084	T_{14} (A’)	$^3MC_{x^2-y^2}$	3.56	348	0.0000

Table S9. Electronic characters as visualized by charge density differences (CDDs) for singlet-singlet and singlet-triplet transitions obtained of **[Fe-mpz⁺]** as obtained at the TDDFT level of theory (B3LYP/def2-SVP). Solvent (water) effects were considered by a polarizable continuum model (SMD). Charge transfer takes place from red to blue.

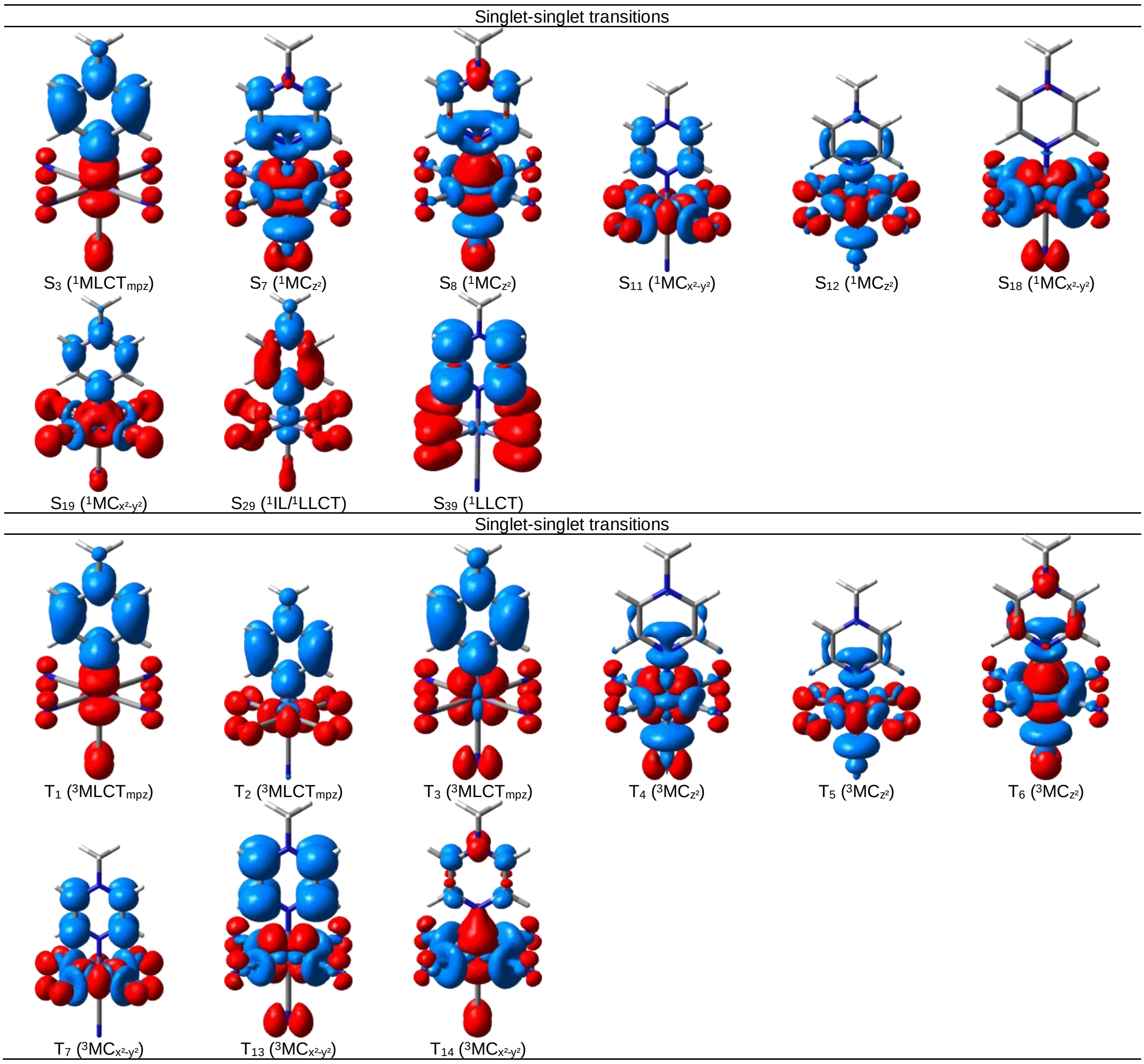

Table S11. Simulated energies (relative to S_0 equilibrium structure) and spin densities of the fully relaxed ^{3}MLCT and ^{3}MC structures of **[Fe-mpz⁺]** and **[Fe-mbpy⁺]** and (approximated) triplet transition state (^{3}TS) along the ^{3}MLCT-^{3}MC relaxation pathway as obtained at the unrestricted DFT level of theory (B3LYP/def2-SVP). Solvent (water) effects were considered by a combination of explicit (10 H_2O molecules) and implicit solvent effects (SMD). Charge transfer takes place from red to blue; spin densities are provided for triplet ground states (T_1).

	**[Fe-mpz⁺]**			**[Fe-mbpy⁺]**		
	**$^3MLCT_{mpz}$**	**^3TS**	**$^3MC_{z^2}$**	**$^3MLCT_{mbpy}$**	**^3TS**	**$^3MC_{z^2}$**
Δ*E* / eV	0.99	1.27	1.16	1.14	1.36	1.16
Spin density	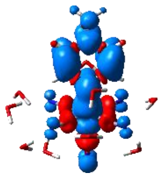	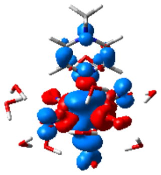	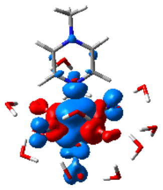	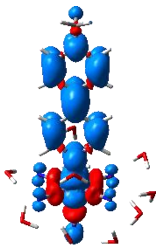	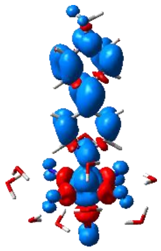	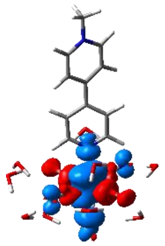

5.5 UV-Vis SEC

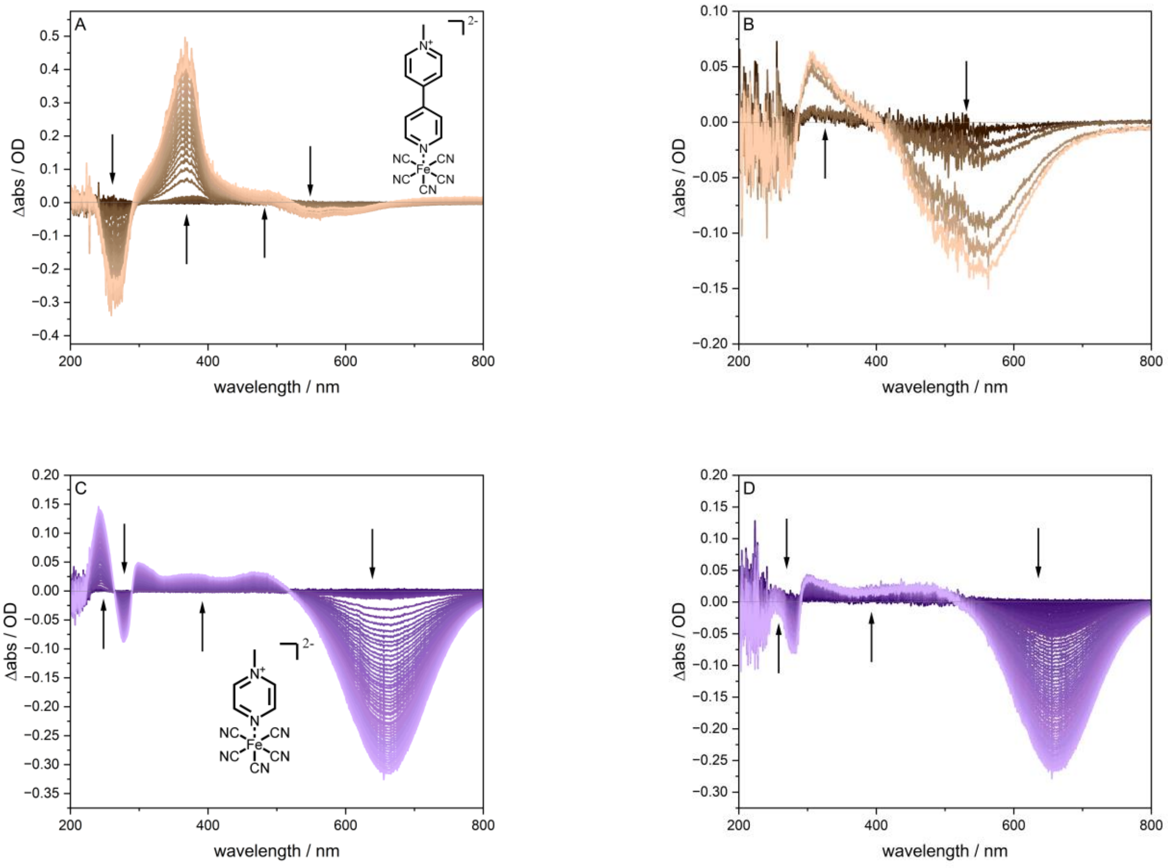

Figure S14. Difference UV-Vis SEC spectra of **[Fe-mbpy⁺]** (A, B) and **[Fe-mpz⁺]** (C, D) during the first reduction (A -0.7V, C -0.7V) and the first oxidation (B +0.7 V, D +0.8V)

5.6 Photodecomposition

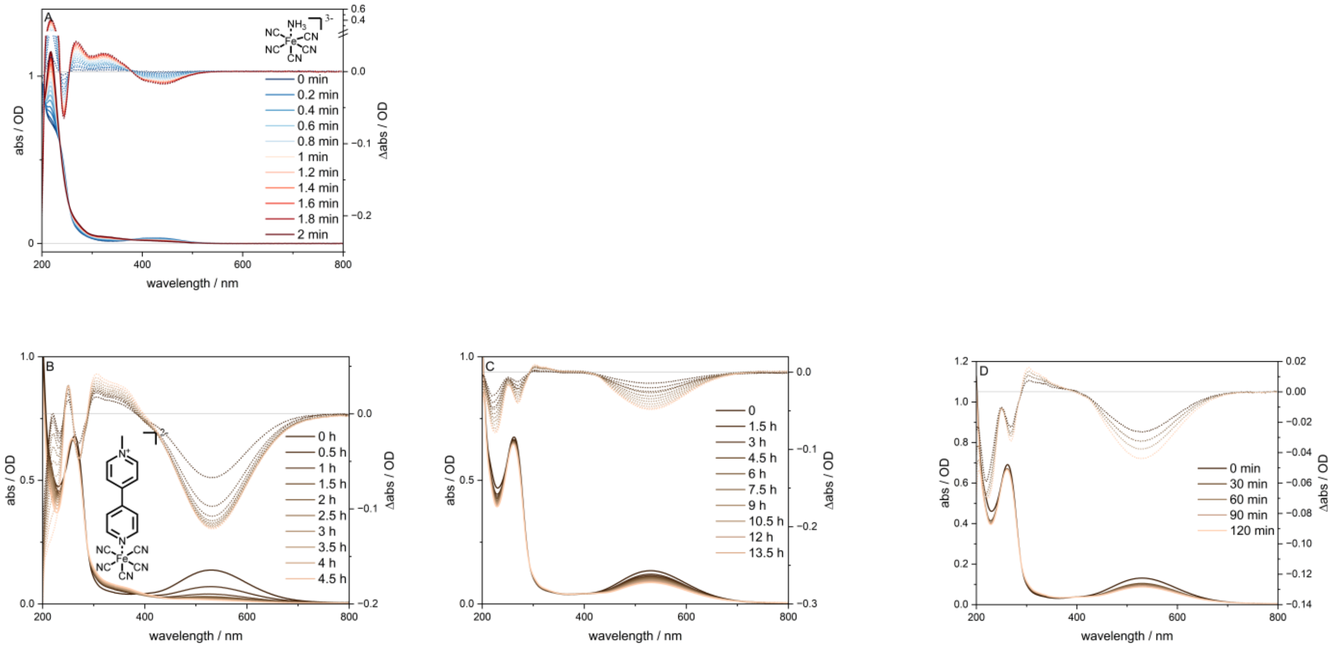

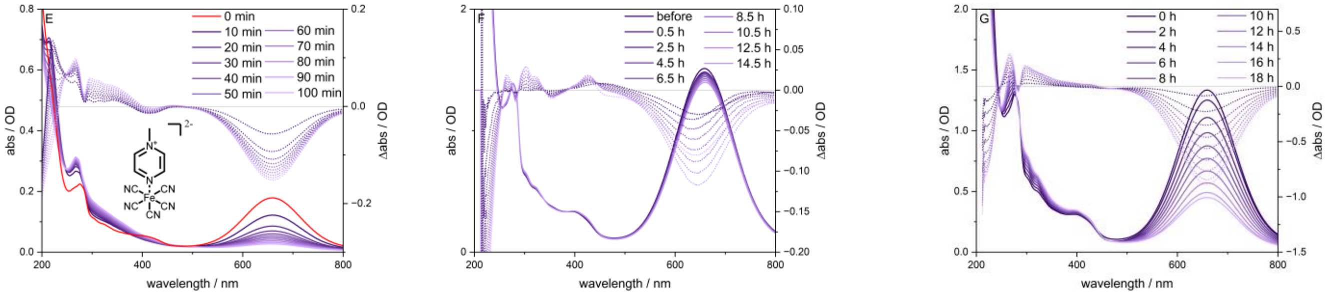

Figure S15. Illumination studies of **[FeNH$_3$]**, **[Fe-mbpy$^+$]** and **[Fe-mpz$^+$]** with 405 nm (A, B, E), 590 nm (C), 660 nm (F) and 5500 K white-light (D, H) illumination. Concentrations: A: 165 µM, B, D, E: 33µM, C, F, G: 330 µM. See experimental details for further information. Dotted lines show difference spectra

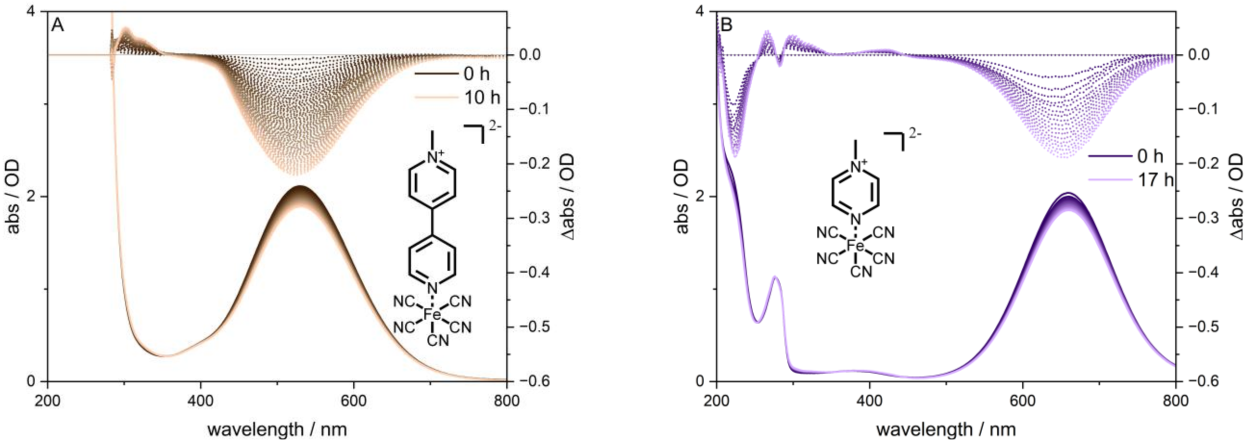

Figure S16. Illumination spectra of concentrated solutions **[Fe-mbpy$^+$]** (A) and **[Fe-mpz$^+$]** B) at 590 nm, respective 660 nm excitation. Values below 300 nm for **[Fe-mbpy$^+$]** have been cut due to detector saturation.

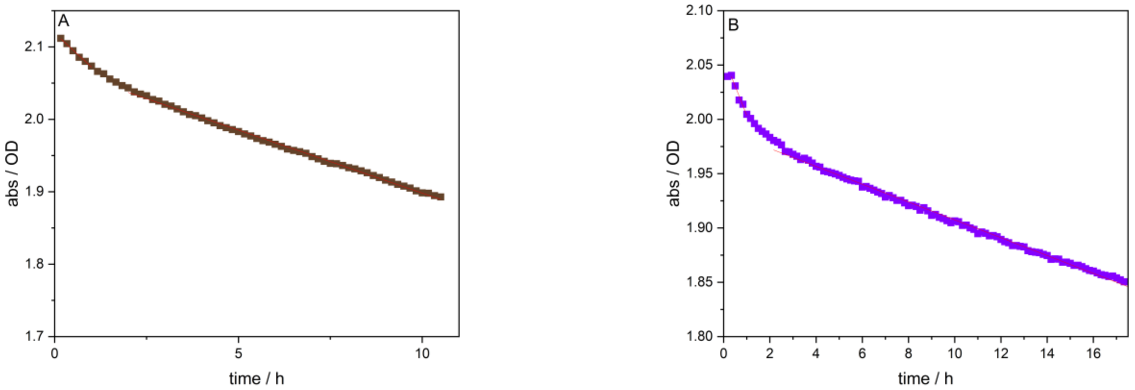

Figure S17. Kinetic traces of **[Fe-mbpy$^+$]** at 530 nm (illumination at 590 nm) (A), and **[Fe-mpz$^+$]** at 660 nm (illumination at 660 nm) (B). See Figure S16 for spectra. Photodissociation quantum yields determined from linear fits in the beginning and in the end were determined as 0.22% (early) and 0.08% (late) for **[Fe-mbpy$^+$]** and 0.044% (early) and 0.0007% (late) for **[Fe-mpz$^+$]**.

6. References

(1) Lutz, F.; Lorenzo-Parodi, N.; Schmidt, T. C.; Niemeyer, J. Heteroternary cucurbit8uril complexes as supramolecular scaffolds for self-assembled bifunctional photoredoxcatalysts. *Chem. Commun.* **2021**, *57* (23), 2887–2890. DOI: 10.1039/D0CC08025J.
(2) Monteiro, M. C.; Toledo, K. C. F.; Pires, B. M.; Wick, R.; Bonacin, J. A. Improvement in Efficiency of the Electrocatalytic Reduction of Hydrogen Peroxide by Prussian Blue Produced from the [Fe(CN) 5 (mpz)] 2– Complex. *Eur. J. Inorg. Chem.* **2017**, *2017* (13), 1979–1988. DOI: 10.1002/ejic.201601540.
(3) Ulusoy Ghobadi, T. G.; Akhuseyin Yildiz, E.; Buyuktemiz, M.; Sadigh Akbari, S.; Topkaya, D.; İsci, Ü.; Dede, Y.; Yaglioglu, H. G.; Karadas, F. A Noble-Metal-Free Heterogeneous Photosensitizer-Relay Catalyst Triad That Catalyzes Water Oxidation under Visible Light. *Angew. Chem. Int. Ed* **2018**, *57* (52), 17173–17177. DOI: 10.1002/anie.201811570.
(4) Coe, B. J.; Jones, L. A.; Harris, J. A.; Brunschwig, B. S.; Asselberghs, I.; Clays, K.; Persoons, A.; Garín, J.; Orduna, J. Syntheses and spectroscopic and quadratic nonlinear optical properties of extended dipolar complexes with ruthenium(II) ammine electron donor and N-methylpyridinium acceptor groups. *J. Am. Chem. Soc.* **2004**, *126* (12), 3880–3891. DOI: 10.1021/ja0315412.
(5) Damrauer, N. H.; McCusker, J. K. Ultrafast Dynamics in the Metal-to-Ligand Charge Transfer Excited-State Evolution of [Ru(4,4'-diphenyl-2,2'-bipyridine) 3] 2+. *J. Phys. Chem. A* **1999**, *103* (42), 8440–8446. DOI: 10.1021/jp9927754.
(6) Seidler, B.; Tran, J. H.; Hniopek, J.; Traber, P.; Görls, H.; Gräfe, S.; Schmitt, M.; Popp, J.; Schulz, M.; Dietzek-Ivanšić, B. Photophysics of Anionic Bis(4H-imidazolato)CuI Complexes. *Chem. Eur. J.* **2022**, *28* (72), e202202697. DOI: 10.1002/chem.202202697.
(7) Müller, C.; Pascher, T.; Eriksson, A.; Chabera, P.; Uhlig, J. KiMoPack: A python Package for Kinetic Modeling of the Chemical Mechanism. *J. Phys. Chem. A* **2022**, *126* (25), 4087–4099. DOI: 10.1021/acs.jpca.2c00907.
(8) Ryseck, G.; Villnow, T.; Hugenbruch, S.; Schaper, K.; Gilch, P. Strong impact of the solvent on the photokinetics of a 2(1H)-pyrimidinone. *Photochem Photobiol Sci* **2013**, *12* (8), 1423–1430. DOI: 10.1039/c3pp50074h.
(9) Frisch, M. J.; Trucks, G. W.; Schlegel, H. B.; Scuseria, G. E.; Robb, M. A.; Cheeseman, J. R.; Scalmani, G.; Barone, V.; Petersson, G. A.; Nakatsuji, H.; Li, X.; Caricato, M.; Marenich, A. V.; Bloino, J.; Janesko, B. G.; Gomperts, R.; Mennucci, B.; Hratchian, H. P.; Ortiz, J. V.; Izmaylov, A. F.; Sonnenberg, J. L.; Williams; Ding, F.; Lipparini, F.; Egidi, F.; Goings, J.; Peng, B.; Petrone, A.; Henderson, T.; Ranasinghe, D.; Zakrzewski, V. G.; Gao, J.; Rega, N.; Zheng, G.; Liang, W.; Hada, M.; Ehara, M.; Toyota, K.; Fukuda, R.; Hasegawa, J.; Ishida, M.; Nakajima, T.; Honda, Y.; Kitao, O.; Nakai, H.; Vreven, T.; Throssell, K.; Montgomery Jr., J. A.; Peralta, J. E.; Ogliaro, F.; Bearpark, M. J.; Heyd, J. J.; Brothers, E. N.; Kudin, K. N.; Staroverov, V. N.; Keith, T. A.; Kobayashi, R.; Normand, J.; Raghavachari, K.; Rendell, A. P.; Burant, J. C.; Iyengar, S. S.; Tomasi, J.; Cossi, M.; Millam, J. M.; Klene, M.; Adamo, C.; Cammi, R.; Ochterski, J. W.; Martin, R. L.; Morokuma, K.; Farkas, O.; Foresman, J. B.; Fox, D. J. *Gaussian 16 Rev. B.01*, 2016.
(10) Becke, A. D. Density‐functional thermochemistry. III. The role of exact exchange. *J. Chem. Phys.* **1993**, *98* (7), 5648–5652. DOI: 10.1063/1.464913.
(11) Lee, C.; Yang, W.; Parr, R. G. Development of the Colle-Salvetti correlation-energy formula into a functional of the electron density. *Phys. Rev. B* **1988**, *37* (2), 785–789. DOI: 10.1103/PhysRevB.37.785.
(12) Marenich, A. V.; Cramer, C. J.; Truhlar, D. G. Universal solvation model based on solute electron density and on a continuum model of the solvent defined by the bulk dielectric constant and atomic surface tensions. *Journal of Physical Chemistry B* **2009**, *113* (18), 6378–6396. DOI: 10.1021/jp810292n.
(13) Grimme, S.; Ehrlich, S.; Goerigk, L. Effect of the damping function in dispersion corrected density functional theory. *J. Comput. Chem.* **2011**, *32* (7), 1456–1465. DOI: 10.1002/jcc.21759.
(14) Zobel, J. P.; Kruse, A.; Baig, O.; Lochbrunner, S.; Bokarev, S. I.; Kühn, O.; González, L.; Bokareva, O. S. Can range-separated functionals be optimally tuned to predict spectra and excited state dynamics in photoactive iron complexes? *Chem. Sci.* **2023**, *14* (6), 1491–1502. DOI: 10.1039/D2SC05839A.
(15) Wegeberg, C.; Häussinger, D.; Kupfer, S.; Wenger, O. S. Controlling the Photophysical Properties of a Series of Isostructural d6 Complexes Based on Cr0, MnI, and FeII. *J. Am. Chem. Soc.* **2024**, *146* (7), 4605–4619. DOI: 10.1021/jacs.3c11580.
(16) Mennucci, B.; Cappelli, C.; Guido, C. A.; Cammi, R.; Tomasi, J. Structures and properties of electronically excited chromophores in solution from the polarizable continuum model coupled to the time-dependent density functional theory. *J. Phys. Chem. A* **2009**, *113* (13), 3009–3020. DOI: 10.1021/jp8094853.
(17) Pracht, P.; Bohle, F.; Grimme, S. Automated exploration of the low-energy chemical space with fast quantum chemical methods. *Phys. Chem. Chem. Phys.* **2020**, *22* (14), 7169–7192. DOI: 10.1039/C9CP06869D.
(18) Shillito, G. E.; Hall, T. B. J.; Preston, D.; Traber, P.; Wu, L.; Reynolds, K. E. A.; Horvath, R.; Sun, X. Z.; Lucas, N. T.; Crowley, J. D.; George, M. W.; Kupfer, S.; Gordon, K. C. Dramatic Alteration of 3ILCT Lifetimes Using Ancillary Ligands in Re(L)(CO)3(phen-TPA) n+ Complexes: An Integrated Spectroscopic and Theoretical Study. *J. Am. Chem. Soc.* **2018**, *140* (13), 4534–4542. DOI: 10.1021/jacs.7b12868.
(19) Sutton, J. J.; Preston, D.; Traber, P.; Steinmetzer, J.; Wu, X.; Kayal, S.; Sun, X.-Z.; Crowley, J. D.; George, M. W.; Kupfer, S.; Gordon, K. C. Excited-State Switching in Rhenium(I) Bipyridyl Complexes with Donor-Donor and Donor-Acceptor Substituents. *J. Am. Chem. Soc.* **2021**, *143* (24), 9082–9093. DOI: 10.1021/jacs.1c02755.
(20) Mengele, A. K.; Müller, C.; Nauroozi, D.; Kupfer, S.; Dietzek, B.; Rau, S. Molecular Scylla and Charybdis: Maneuvering between pH Sensitivity and Excited-State Localization in Ruthenium Bi(benz)imidazole Complexes. *Inorg. Chem.* **2020**, *59* (17), 12097–12110. DOI: 10.1021/acs.inorgchem.0c01022.
(21) Kupfer, S. *Quantum Chemical Data - A Heterodox Approach for Designing Iron Photosensitizers: Qunatum Chemical Data - Pentacyanoferrate Complexes with Monodentate Pyridinium based Acceptor Ligands*. https://doi.org/10.5281/zenodo.11519056.

7. Author Contributions

	Schmidt	Chalil Oglou	Tuncer	Ulusoy Ghobadi	Tekir	Ozvural Sertcelik	El-Neny	Doehler	Ozcubukcu	Kupfer	Dietzek-Ivansic	Karadas
Conceptualization	X	X		X						X	X	X
Data curation	X	X	X		X			X	X	X	X	X
Formal analysis	X	X	X	X	X	X	X	X	X	X	X	X
Funding acquisition											X	X
Investigation	X	X	X	X	X	X	X	X		X		
Methodology	X	X		X						X		X
Project admin										X	X	X
Resources									X	X	X	X
Software	X	X								X		

Supervision									X	X	X	X
Validation	X	X	X	X						X	X	X
Writing – original draft	X	X	X						X	X	X	X
Writing – review + editing	X								X	X	X	X
